# Fluorescent risedronate analogue 800CW-pRIS improves tooth extraction-associated abnormal wound healing in zoledronate-treated mice

**DOI:** 10.1038/s43856-022-00172-x

**Published:** 2022-09-05

**Authors:** Hiroko Okawa, Takeru Kondo, Akishige Hokugo, Philip Cherian, Oskar Sundberg, Jesus J. Campagna, Boris A. Kashemirov, Varghese John, Shuting Sun, Frank H. Ebetino, Charles E. McKenna, Ichiro Nishimura

**Affiliations:** 1 Weintraub Center for Reconstructive Biotechnology, Division of Regenerative & Reconstructive Sciences, UCLA School of Dentistry, Los Angeles, CA 90095 USA; 2 Division of Molecular and Regenerative Prosthodontics, Tohoku University Graduate School of Dentistry, Sendai, 980-8575 Japan; 3 Regenerative Bioengineering and Repair Laboratory, Division of Plastic and Reconstructive Surgery, David Geffen School of Medicine at UCLA, Los Angeles, CA 90095 USA; 4 BioVinc LLC, Pasadena, CA 91107 USA; 5 Department of Chemistry, University of Southern California, Los Angeles, CA 90089 USA; 6 Department of Neurology, David Geffen School of Medicine at UCLA, Los Angeles, CA 90095 USA

**Keywords:** Drug development, Drug delivery

## Abstract

**Background:**

Bisphosphonate-related osteonecrosis of the jaw (BRONJ) is a rare but serious side effect of nitrogen-containing bisphosphonate drugs (N-BPs) frequently prescribed to reduce skeletal-related events in bone malignancies and osteoporosis. BRONJ is associated with abnormal oral wound healing after dentoalveolar surgery and tooth extraction. We previously found that N-BP chemisorbed to bone mineral hydroxyapatite was dissociated by secondary applied N-BP. This study investigated the effect of the surface equilibrium-based removal of N-BP from jawbone on tooth extraction wound healing of zoledronate (ZOL)-treated mice.

**Methods:**

A pharmacologically inactive N-BP derivative (the 4-pyridyl isomer of risedronate equipped with a near-infrared 800CW fluorescent imaging dye, 800CW-pRIS) was designed and synthesized. 800CW-pRIS was intra-orally injected or topically applied in a deformable nano-scale vesicle formulation (DNV) to the palatal tissue of mice pretreated with ZOL, a potent N-BP. The female C56BL6/J mice were subjected to maxillary molar extraction and oral wound healing was compared for 800CW-pRIS/ZOL, ZOL and untreated control groups.

**Results:**

800CW-pRIS is confirmed to be inactive in inhibiting prenylation in cultured osteoclasts while retaining high affinity for hydroxyapatite. ZOL-injected mice exhibit delayed tooth extraction wound healing with osteonecrosis relative to the untreated controls. 800CW-pRIS applied topically to the jaw one week before tooth extraction significantly reduces gingival oral barrier inflammation, improves extraction socket bone regeneration, and prevents development of osteonecrosis in ZOL-injected mice.

**Conclusions:**

Topical pre-treatment with 800CW-RIS in DNV is a promising approach to prevent the complication of abnormal oral wound healing associated with BRONJ while retaining the anti-resorptive benefit of legacy N-BP in appendicular or vertebrate bones.

## Plain Language Summary

Bisphosphonates are valuable drugs for bone malignancies and osteoporosis, but in some cases, cancer patients taking bisphosphonates have experienced abnormal wound healing after oral surgery, a condition referred to as bisphosphonate-related osteonecrosis of the jaw (BRONJ). Although BRONJ is relatively uncommon, the lack of effective therapies to prevent or treat this serious condition has been a concern. We report that mice pretreated with a bisphosphonate drug and then subjected to extraction of a tooth display abnormal wound healing. This can be prevented by topical pretreatment with an inactive bisphosphonate called 800CW-pRIS. These results provide support for a new approach to address the problem of BRONJ, that might preserve the benefits of bisphosphonate drug treatment while minimizing side effects.

## Abstract (web summary)

Okawa et al. evaluate a fluorescent risedronate analogue, 800CW-pRIS, as a potential treatment for medication-related osteonecrosis of the jaw. The authors demonstrate an improvement in wound healing and inflammation with 800CW-pRIS pre-treatment in a mouse model of bisphosphonate-associated osteonecrosis following tooth extraction.

## Introduction

Nitrogen-containing bisphosphonates (N-BPs) are prototypical antiresorptive agents and effective drugs for treating abnormal osteolysis associated with tumors residing in and metastasizing to bone marrow, such as multiple myeloma and breast cancer, respectively^1–3^. At a lower dose, N-BPs have also been widely used to reduce the risk of osteoporotic bone fractures^4^. N-BPs are synthetic analogues of pyrophosphate, which plays an important role in bone metabolism and cholesterol biosynthesis. Through the mevalonate pathway, isopentenyl pyrophosphate (IPP) is converted to farnesyl pyrophosphate (FPP) by FPP synthase (FPPS). Pharmacologically active N-BPs such as risedronate (RIS) and zoledronate (ZOL) occupy the allylic substrate binding pocket of FPPS, effectively preventing the synthesis of FPP from IPP^5–7^. As a result, osteoclasts that internalize N-BP exhibit poor prenylation of the downstream proteins, leading to abnormal cytoskeleton formation and premature detachment from the bone resorption lacunae^8^.

N-BPs have been shown to prevent skeletal-related events (SRE) and decrease bone pain in cancer patients, with few serious side effects^9,10^. However, the FDA adverse event reporting system (FAERS) has posted clinical reports of oral complications collectively known as medication-related osteonecrosis of the jaw (MRONJ)^11^. The American Association of Oral & Maxillofacial Surgeons (AAOMS) defined MRONJ as an unhealed oral wound with exposed jawbone or fistula reaching to the jawbone surface in patients with a history of antiresorptive medications, including N-BPs and humanized anti-receptor activator of nuclear factor-κB ligand (RANKL) monoclonal antibody (Denosumab), as well as of angiogenesis inhibitors^12^. The oral side effect of anti-resorptive agents in the FAERS database peaked from the first quarter of 2010 to the first quarter of 2014 with ~30,000 cases during this period; N-BP comprised the largest segment of reported adverse events^11^. Widespread concern about the rare but often severe oral complications is believed to have contributed to markedly decreased acceptance of N-BPs by osteoporosis patients, despite the well-established benefit of these drugs for this indication^13^. Currently, there is no established and effective preventative treatment or therapy for N-BP-related oral complications.

**Fig. 1:**
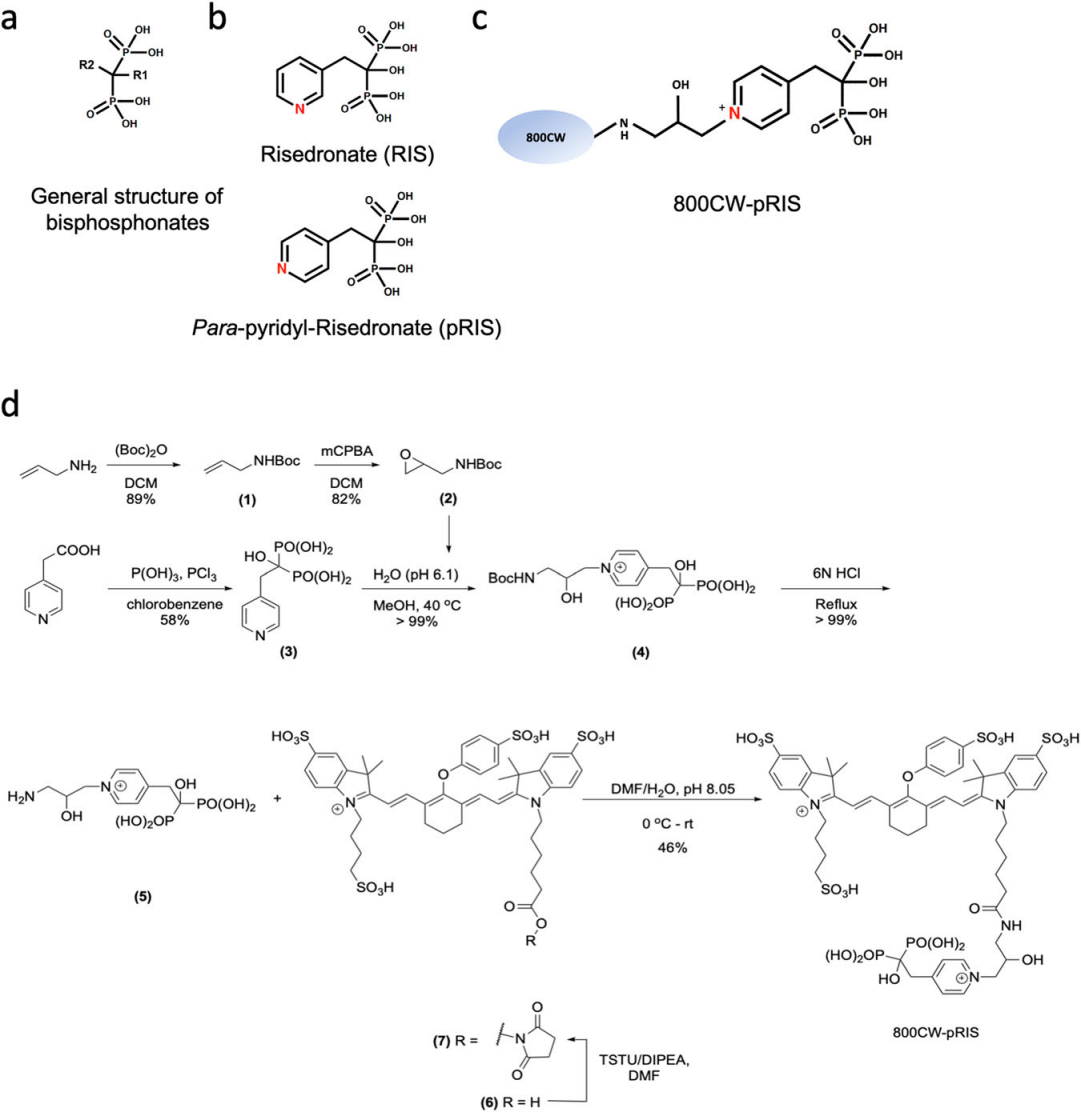
Chemical structure and synthesis of 800CW-pRIS. **a** General structure of bisphosphonates. **b** Chemical structure of risedronate (RIS) and *para*-pyridyl-risedronate (pRIS). **c** Chemical structure of 800CW-pRIS. **d** Synthesis scheme of 800CW-pRIS (anionic counterions are omitted for clarity).

We have previously reported that N-BP adsorption to bone minerals is not permanent but rather establishes an equilibrium^14,15^. Thus, we hypothesized that the legacy N-BP adsorbed on the jawbone might be displaced and replaced by a pharmacologically inactive N-BP through the equilibrium-based molecular competitive binding, leading to the attenuation of abnormal oral wound healing. The chemical structure of N-BPs acutely determines their antiresorptive potency. Ca^2+^ ion chelation by the bidentate phosphonate moieties of the BP structure abetted, if present, by an α-hydroxy side chain (Fig. 1a) facilitates a high affinity to bone mineral, which is primarily hydroxyapatite (HAp)^16^. After adsorption to the bone surface, N-BPs are thought to remain quiescent until osteoclastic bone resorption. Steady-state release of adsorbed N-BP drugs from bone is slow, as shown by studies determining their half-lives in the skeleton, which range from months to several years^17^. However, we have shown that N-BPs can be rapidly displaced from synthetic HAp or bone mineral surfaces by the application of competing BPs in solution^14,15^. This led us to explore the idea that displacement of legacy N-BP from the jawbone by local treatment with a pharmacologically inactive N-BP prior to the high-risk dentoalveolar surgical manipulations would abate the development of abnormal wound healing.

Holtmann et al. reviewed the literature and reported that the majority of animal models for bisphosphonate-related ONJ (BRONJ) were laboratory rodents: rats (52.3%) and mice (34.1%)^18^. Those rodent models from different laboratories follow a similar protocol: N-BP systemic injection and tooth extraction. The complications associated with dentoalveolar surgery and tooth extraction have been reported as a characteristic disease phenotype of BRONJ^19^, which was similarly observed in the rodent tooth extraction models^20–22^. Human patients show various confounding factors that may influence the development of MRONJ/BRONJ. Recently, modified rodent models have been developed to evaluate the role of these confounding factors such as autoimmune diseases^23^ and periodontitis^24^ on the severity of BRONJ lesions. However, the pathological mechanism of BRONJ has not yet been fully established.

The present study hypothesized that the presence of N-BP on the jawbone played an important role in oral wound complications. Here we describe the evaluation of this hypothesis in the tooth extraction wound healing of an N-BP-treated murine model, using as the displacing agent: a novel fluorescent dye-conjugated RIS analogue, 800CW-pRIS, modified to remove its inherent anti-resorptive activity. The newly synthesized 800CW-pRIS lacked pharmacological function while maintaining a strong affinity to hydroxyapatite and displaced pretreated ZOL. In ZOL-pretreated mice, topical application of 800CW-pRIS to oral mucosa attenuated abnormal tooth extraction socket wound healing and jawbone osteonecrosis development.

## Methods

### Chemical reagents

IRDye® 800CW-carboxylate was purchased from Licor Biosciences. *N,N,N,N*-tetramethyl-*O*-(*N*-succinimidyl)uronium tetrafluoroborate (TSTU) was purchased from Oakwood Chemical. All other reagents were purchased from either Sigma-Aldrich or Alfa Aesar. Allylamine was distilled under N_2_; CH_2_Cl_2_ was distilled from P_2_O_5_. All other reagents were used as supplied by the manufacturer. Anhydrous *N*,*N*-dimethylformamide (DMF), tetrahydrofuran (THF), dichloromethane (DCM), chlorobenzene, acetonitrile, methanol, ethanol, ether, and diisopropylethylamine (DIPEA) were purchased from EMD Millipore Corporation or VWR. Sodium carbonate, sodium bicarbonate, phosphoric acid, magnesium sulfate, sodium hydroxide, and hydrochloric acid were purchased from Fisher Scientific. ZOL was obtained from UCLA Pharmacy (Reclast®, Novartis, Basel, Switzerland). DOTAP (1,2-dioleoyloxy-3-(trimethylammonium)propane sulfate), DPPC (diphosphatidylcholine), CH (cholesterol) and a nonionic surfactant (Span 80) were acquired from Sigma-Aldrich (St. Louis, MO).

### Ethics statement

All experimental protocols using animals were reviewed and approved by the UCLA Animal Research Committee (ARC#1997-136) and followed the PHS policy for Humane Care and use of Laboratory Animals and UCLA Animal Care Use Training Manual guidelines.

### Design of 800CW-conjugated *para*-pyridyl-risedronate (800CW-pRIS)

The position of nitrogen in the pyridine ring of RIS is an important determinant of its antiresorptive activity^25^. When the 3-nitrogen of RIS is relocated to the 4-position (i.e., from *meta* to *para*, pRIS; Fig. 1b), antiresorptive activity is reduced while the high affinity of the BP moiety to HAp is retained^26,27^. We further modified pRIS by conjugation to a near-infrared dye, IRDye 800CW (Fig. 1c).

### Synthesis of 800CW-pRIS (Fig. 1d)

#### *tert*-Butyl allylcarbamate^28^ (1)

A solution of di-*tert*-butyl dicarbonate (2.18 g, 10 mmol) in dry DCM (7 mL) was added dropwise to an ice-cold solution of allylamine (0.57 g, 10 mmol) in dry DCM (3.5 mL). The solution was brought to room temperature and stirred for 4 h. The reaction mixture was then diluted with additional DCM (10 mL) and extracted with 5% citric acid solution, followed by brine. The organic layer was dried over Na_2_SO_4_ and concentrated under vacuum to provide 1.4 g (89%) of *tert*-butyl allylcarbamate **(1)** as a yellow oil which was used in next step without further purification. ^1^H NMR (300 MHz, CDCl_**3**_): δ 5.92−5.75 (m, 1H), 5.24−5.06 (m, 2H), 4.59 (br, 1H), 3.74 (br, 2H), 1.44 (s, 9H).

#### *tert*-Butyl (oxiran-2-ylmethyl)carbamate^29^ (2)

To an ice-cold solution of **1** (1.4 g, 8.90 mmol) in DCM (50 mL) was gradually added *meta*-chloroperoxybenzoic acid (mCPBA) (3.07 g, 17.81 mmol). The solution was brought to rt and stirred for 20 h, then diluted with DCM (50 mL) and sequentially extracted with 75 mL each of 10% Na_2_SO_3_, saturated NaHCO_3_ (3x), water, and brine. The DCM layer was dried over MgSO_4_ and concentrated under vacuum to provide 1.27 g (82%) of **2** as a light-yellow oil which was used in next step without further purification. ^1^H NMR (300 MHz, CDCl_**3**_): δ 4.74 (br, 1H), 3.60−3.42 (m, 1H), 3.25−3.13 (m, 1H), 3.12−3.01 (m, 1H), 2.76 (t, *J* = 4.8 Hz, 1H), 2.53 (dd, *J* = *4.7, 2.7* Hz, 1H), 1.44 (s, 9H).

#### (1-Hydroxy-2-(pyridin-4-yl)ethane-1,1-diyl)bis(phosphonic acid)^30^ (3)

A 500 ml round bottom flask was charged with 4-pyridylacetic acid hydrochloride (2.60 g, 15 mmol), phosphorous acid (3.68 g, 45 mmol), and chlorobenzene (75 mL). The mixture was heated to 100 °C and phosphorus trichloride (6.18 g, 45 mmol) was added along the sides of the flask using a syringe. A reflux condenser was attached and heating continued at 100 °C for 3 h. A yellow gummy oil formed during the course of the reaction. After cooling to rt and then 0 °C, excess chlorobenzene was decanted and aq. 3 N HCl (125 mL) was added to the residual yellow oil. The flask was refitted with a reflux condenser and heated at 100 °C overnight. The solvent was then evaporated under vacuum, the residue was suspended in warm water (75 mL) and methanol was added until the solution became turbid. After reflux for 1 h, the precipitated product was filtered, washed with EtOH, and dried under high vacuum to provide 2.58 g (58%) of **3** as a white solid. ^1^H NMR (300 MHz, D_2_O + Na_2_CO_3_): δ 8.31 (d, *J* = 5.88 Hz, 2H), 7.72 (d, *J* = 6.12, 2H), 3.26 (t, *J* = 12.42, 1H). ^31^P NMR (121 MHz, D_2_O + Na_2_CO_3_): δ 16.01 (s).

#### 1-(3-((*tert*-Butoxycarbonyl)amino)−2-hydroxypropyl)−4-(2-hydroxy-2,2-diphosphonoethyl)pyridin-1-ium (4)

To (1-hydroxy-2-(pyridin-4-yl)ethane-1,1-diyl)bis(phosphonic acid) (**3**) (142 mg, 0.5 mmol) was added H_2_O (2 mL) and the pH of the mixture was adjusted to 6.1 using 1 M NaOH. Next, **2** (0.086 g, 0.5 mmol) was dissolved in a minimum volume of MeOH (200 mL) and added and the reaction mixture was heated at 40 °C. The progress of the reaction was monitored by ^31^P NMR. After 16 h, conversion to product was 70% complete and an additional 1/3 equivalent of **2** (0.03 mg, 0.16 mmol) was added with continued stirring at 40 °C. This step was repeated several times, until all of the starting material (**3**) was consumed (62 h). The reaction mixture was filtered and the filtrate was evaporated, leaving a viscous yellow oil. Ether (3 mL) was added, the mixture was sonicated, and the ether was decanted. The residue was then placed under high vacuum to provide 0.269 g (>99%) of **4** as an off-white foam which was used in next step without further purification. ^1^H NMR (300 MHz, D_2_O): δ 8.42 (d, *J* = *6.32* Hz, 2H), 7.92 (d, *J* = 6.32 Hz, 2H), 4.19 (dd*, J* = 14.16, 9.55 Hz, 1H), 4.02−3.91 (m, 1H), 3.39−3.08 (m, 4H), 1.29 (s, 9H). ^31^P NMR (121 MHz, D_2_O): δ 16.12 (s).

#### 1-(3-Amino-2-hydroxypropyl)−4-(2-hydroxy-2,2-diphosphonoethyl)pyridin-1-ium (5)

Intermediate **4** (0.269 g, 0.51 mmol) was refluxed in 6 N HCl (8 mL) for 6 h. The solvent was evaporated, leaving a light-yellow gum which was sonicated in acetonitrile—MeOH to provide 275 mg (>99%) of the product **5** as an off-white solid which was used in next step without further purification. ^1^H NMR (300 MHz, D_2_O): δ 8.50 (d, *J* = 5.45 Hz, 2H), 7.93 (d, *J* = 5.81 Hz, 2H), 4.36−4.13 (m, 2H), 3.36 (t, *J* = 12.10 Hz, 2H), 3.26−3.16 (m, 2H, obscured by MeOH peak), 3.05−2.81 (m, 1H). ^31^P NMR (121 MHz, D_2_O): δ 16.44 (s).

### Characterization of intermediate compounds 4 and 5 (Fig. S1)

For the synthesis of **4**: 0.5 mmol of **3** is converted to **4** with a >99% yield. The stated weight of the obtained product **4** is 0.269 g. The pH at which the reaction is performed is stated to be 6.1. At this pH, MarvinSketch calculations suggest the compound should exist as a trisodium salt. The MW of **4** as a trisodium salt is 522.05 g/mol, which is close to the MW of the compound obtained (0.269 g/0.5 mmol = 538 g/mol). It could be stated that this intermediate was obtained as a trisodium salt. In the following conversion to **5**, 0.269 g, 0.51 mmol of starting material (**4**) is used. If **4** is a trisodium salt, then this number is valid.

The conversion to **5** is an HCl mediated Boc-deprotection. It is reported that 275 mg of compound is obtained in ~99% yield. The MW of **5** as a dichloride salt is 428.01 g/mol. If we obtained 0.51 mmol of **5** as a dichloride salt, then this would result in a mass of 218.3 mg. Therefore, we believe that this compound contains NaCl impurities. Two NaCl per product molecule would result in a pseudo MW of 543.92 g/mol which is close to the calculated MW of the compound at 539.2 g/mol. The presence of NaCl impurities should be properly assessed. As such, we have estimated that **4** is a trisodium salt and also contains one H_2_O, while **5** is a dichloride salt with some sodium chloride impurities.

#### 800CW-pRIS

To an ice-cold mixture of IRDye® 800CW-carboxylate (**6**) (Licor Biosciences, Lincoln, NE) (0.05 g, 0.045 mmol) and DIPEA (0.071 mL, 0.41 mmol) in dry DMF (2 mL), kept under N_2_ and in the dark, was added a solution of TSTU (0.02 g, 0.068 mmol) in DMF (200 mL). After 3 h, TLC (DCM/MeOH, 3:2) showed disappearance of the **6** spot and formation of the 800CW-NHS ester (**7**). The solvent was removed under vacuum and the residue was dissolved in DMF (750 mL), then added to a solution of **5** (0.11 g, 0.22 mmol) in H_2_O (2.5 mL) which was adjusted to pH 8.33 using Na_2_CO_3_ while constantly maintained in the dark at rt. The addition of the 800CW-NHS ester decreased the pH of the reaction mixture to 7.46. The pH was readjusted to 8.05 using Na_2_CO_3_ and the reaction mixture was stirred at rt overnight. HPLC analysis of the reaction mixture after 18 h confirmed formation of the desired product (800CW-pRIS), which was isolated by prep. RP-HPLC using a Phenomenex Luna® 5 µm C18(2) 100 Å, 250 × 21.2 mm, AXIA™ Packed LC Column using A = 0.1 M TEAAc/20%MeOH (pH = 5.0− 5.3) and B = 0.1 M TEAAc/70% MeOH (pH = 5.0−5.3). HPLC Method: 0% B (0–7 min), 0–100% B (7–25 min), 100% B (25–30 min). 800CW-pRIS eluted at ~21 min and was obtained as a triethylammonium salt in 46% yield (free acid basis), product yields were determined by absorbance at 775 nm (PBS buffer, pH 7.4, *ε* = 242,000 M^−1^cm^−1^)^31^. ^1^H NMR (500 MHz, D_2_O): δ 8.46 (d, *J* = *6.86* Hz, 2H), 7.93 (d, *J* = 6.82 Hz, 2H), 7.69 (d, *J* = 9.01 Hz, 2H), 7.67−7.56 (m, 6H), 7.12−7.04 (m, 4H), 5.96 (t, *J* = 14.03 Hz, 2H), 4.59−4.54 (m, 1H), 4.18 (dd, *J* = 14.21, 9.86 Hz, 1H), 4.00−3.95 (m, 1H), 3.83 (dt, *J* = 24.61, 7.27 Hz, 3H), 3.35 (t, *J* = 12.28 Hz, 2H), 3.23−3.14 (m, 2H), 2.91 (q, *J* = 14.54, 7.11 Hz, 1H), 2.78 (t, *J* = *7.43* Hz, 2H), 2.50 (br t, *J* = *6.15* Hz, 3H), 2.09 (t, *J* = 6.94 Hz, 2H), 1.80 (s, 21H), 1.75−1.63 (m, 4H), 1.59−1.51 (m, 2H), 1.47−1.39 (2H). ^31^P NMR (121 MHz, D_2_O): δ 15.82 (s). HRMS (ESI-TOF) *m/z* calcd for C_56_H_70_N_4_O_22_P_2_S_4_ [M-2H]^−2^ 669.1342; found: 669.1351. The characterization of 800CW-pRIS was presented in Fig. S2 (HPLC purity); Fig. S3 (HRMS analysis); Fig. S4 (^1^H NMR spectrum); Fig. S5 (^31^P NMR spectrum); Fig. S6 (UV-VIS absorption and fluorescent emission spectra).

### Displacement of 5-FAM-ZOL by 800CW-pRIS in vitro

Synthetic apatite (carbonate apatite) coated cell culture wells (Bone resorption assay plate 24, Cosmo Bio Co. Ltd, Tokyo, Japan) were incubated with fluorescent-tagged ZOL (10 µM 5-FAM-ZOL in 500 µl MilliQ-treated water: MQW) overnight at 37 °C, 2% CO_2_. After three washes with Milli-Q treated pure water (MQW) for 10 min each, the 5-FAM-ZOL coated wells were then incubated with 10 µM 800CW-pRIS in 500 µl MQW for 2 h at 37 °C, 2% CO_2_ followed by three washes with MQW. One group of wells (*n* = 3) were further incubated by the second application of 10 µM 800CW-pRIS in 500 µl MQW followed by 3 washes using 500 µl MQW for 10 min each. The control wells were incubated with MQW. The fluorescent signal of each well (apatite discs) and recovered solutions after each treatment (either incubating with 5-FAM-ZOL, wash or challenged by 800CW-pRIS) were evaluated (IVIS Lumina II, PerkinElmer, Waltham, MA) using the preset conditions for GFP and 800CW. The experiment was triplicated, and Student’s *t* test was used to compare the 5-FAM-ZOL treated group to each of other groups.

### In silico modeling

In silico docking experiments were performed using the AutoDock Vina software, and ligands were prepared using AutoDockTools 1.5.6^32^. The crystal structure of human FPPS (PDB 1YV5) was used. To verify the validity of our docking method, we compared the RMSD between the docked RIS ligand with the crystallographic RIS pose. We calculated a RMSD of 1.67 Å which is below the commonly used RMSD ≤ 2 cutoff^33^. To assess the pharmacological efficacy of RIS and its analogues we compared their calculated binding energies and to investigate the reasons for the observed differences in binding energies we determined the hydrogen bonding distance and angle between the protonated pyridyl protonated N hydrogen and the T201 oxygen and  the K200 carbonyl oxygen. To force bulky 800CW-pRIS into the relevant FPPS active site, we built the molecule from within the site using ICM-Pro 3.2.

### In vitro osteoclast resorption pit assay

Synthetic apatite (carbonate apatite) coated cell culture wells were incubated with 10 µM ZOL in 500 µl MQW or 10 µM 800CW-pRIS in 500 µl MQW for overnight at 37 °C, 2% CO_2_ followed by extensive washes using 500 µl MQW. Some ZOL-preincubated wells were further incubated with 10 µM 800CW-pRIS in 500 µl MQW once or twice. Control wells were treated with 500 µl MQW. To all wells, RAW274.1 cells (2.5 × 10^4^ cells per well) were inoculated in culture medium supplemented by mouse recombinant receptor activator of nuclear kappa-B ligand (RANKL; Sigma-Aldrich) (100 ng/ml) and incubated at 37 °C, 2% CO_2_. The culture medium was changed after 3 days. After 6 days of incubation the cells were removed with 0.25% Trypsin and the resorption pit generated in the synthetic apatite was photographed and measured using a Java-based image processing program (ImageJ, NIH, Bethesda, MD). This experiment was performed in triplicated wells per group.

### Tooth extraction of zoledronate (ZOL)-pretreated mice

The ZOL-pretreated mouse model has been previously reported in the literature^21,22^. Female C57Bl6/J(B6) mice with an age of eight to ten weeks were purchased from Jackson Laboratory (Bar Harbor, ME) and maintained in the vivarium of the Division of Laboratory Animal Medicine at UCLA with free access to food and water. The mice were lightly anesthetized by isoflurane and ZOL (500 µg/Kg in medical grade saline: 400 µM, 100 µl) was administered as a bolus IV injection through the retro-orbital venous plexus. Seven days after ZOL injection, the maxillary left first molar was extracted using a dental explorer and analgesics (2 mg/Kg Carprofen) was provided every 12 h for 2 days^21,22^. The only mice that tooth extracted without remaining root were included in the study. To avoid food impaction, the mice were fed a gel diet (DietGel® Recovery, ClearH2O, Westbrook, ME) for the first 7 days. The mice were euthanized after 14 days of tooth extraction by 100% CO_2_ inhalation and the maxillae were harvested.

### 800CW-pRIS administration by local intra-oral injection

The intra-oral injection of 800CW-pRIS (100 µM, 2 µl) to mouse palatal tissue using Hamilton syringe with a 33-gauge needle under a surgical microscope^14^ was first examined for 800CW fluorescent signal 3 days after the injection by the in vivo imaging system with 740 nm emission (IVIS Lumina III, Perkin Elmer, Waltham, MA).

We have previously used the mouse model described above to examine the effect of intra-oral injection of non-nitrogen containing BP for the attenuation of abnormal or delayed tooth extraction wound healing of ZOL-treated mice^14^, based on which we determined the number of mice per group to be *n* = 8 by the preliminary results. The primary outcome measure was not used to determine the sample size.

Four days after the ZOL IV injection, ZOL-pretreated mice were randomly divided into two groups. One group received an intra-oral injection of 2 µl of 100 µM 800CW-pRIS and the other group received an intra-oral injection of 2 µl of 0.9% saline as a negative control (*n* = 8 per group, *n* = 16 in total). After the mice were anesthetized by isoflurane, intra-oral injection to the palatal gingiva adjacent to the left first molar was performed. The maxillary left first molar was extracted 3 days after the 800CW-pRIS intra-oral injection and the mice were euthanized after 14 days of tooth extraction by 100% CO_2_ inhalation. The maxilla containing the tooth extraction wound was harvested and photographed. The maxilla tissue was then fixed in 10% buffered formalin and subjected to micro-CT imaging followed by EDTA-decalcification and conventional paraffin-embedded histological section preparation for outcome assessments (see below).

### 800CW-pRIS-DNV synthesis

800CW-pRIS-DNV were synthesized using established protocols^34^. Briefly, a lipid mixture containing diphosphatidylcholine (DPC), cholesterol (CH), 1,2-dioleoyloxy-3-(trimethylammonium)propane-sulfate (DOTAP) for the positive surface charge or 1,2-dipalmitoyl-*sn*-glycero-3-phosphocholine (DMPC) for the negative surface charge, was dissolved in isopropyl alcohol and Span80 (15% v/v) was added. 800CW-pRIS was dissolved in pure water. The aqueous and organic solutions were injected into the microfluidic reactor and the positive charge-DNV and negative charge-DNV were synthesized.

### Characterization of 800CW-pRIS-DNV

After the synthesis of 800CW-pRIS-DNV, the size was assessed by dynamic light scattering and the surface charge was assessed by zeta potential measurements using a Zeta sizer instrument (Nano-ZS, Malvern, Worcestershire, UK). Then, samples were dialyzed overnight to remove encapsulated 800CW-pRIS. To calculate encapsulation efficiency of 800CW-pRIS, DNVs were disintegrated by ice-cold acetonitrile followed by water bath sonication. The solution was centrifuged (15 min, 20,000 × *g*) and the supernatant was collected. The absorption of 800CW was determined at 770 nm (SpectraMax M5, Molecular Devices, San Jose, CA). The encapsulation efficiency (EE) was calculated:

$${{{{{\rm{EE}}}}}}=({{{{{\rm{Moles}}}}}}\; {{{{{\rm{calculated}}}}}}\; {{{{{\rm{in}}}}}}\; {{{{{\rm{the}}}}}}\; {{{{{\rm{DNV}}}}}}/{{{{{\rm{Initial}}}}}}\; {{{{{\rm{moles}}}}}}\; {{{{{\rm{used}}}}}}\; {{{{{\rm{in}}}}}}\; {{{{{\rm{the}}}}}}\; {{{{{\rm{synthesis}}}}}})\times 100 \%$$

### Trans-gingival epithelium administration test

To assess the efficacy of trans-gingival administration of 800CW-pRIS-DNV, we used a commercially available cultured gingival tissue model (EpiGingival™, MatTek Corporation, Ashland, MA). This in vitro model consists of human oral epithelial cells cultured on cell culture insert. The cell insert was filled with 300 µl of assay medium. Negatively or positively charged 800CW-pRIS-DNV powder was suspended in MiliQ water to make a 25 µM solution. 100 µl of such 800CW-pRIS-DNV solution was added on the oral epithelial cells in the cell insert and incubated at 37 °C in a 5% CO_2_ humidified incubator, according to the manufacture’s protocol. As a control without DNV, we used 25 µM 800CW-pRIS in MiliQ water. The assay medium was changed every 30 min and measured the absorbance in a spectrophotometer at 774 nm with a micro plate reader (SYHNERGY H1 plate reader). The optical density (OD) measurement per well was described as the cumulative OD data, in which the OD measurement was sequentially added to the OD measurement of the previous time point. From this study, positively charged 800CW-pRIS-DNV was selected for the following in vivo studies.

### 800CW-pRIS-DNV topical application to mouse palatal gingiva

For topical application to mouse gingival tissue, 800CW-pRIS-DNV powder was suspended in MiliQ water to make 100 µM solution. To protect the topical treatment site, a custom-made oral appliance was used to cover the whole palatal tissue for 1 h and then the oral appliance was removed. The delivery of 800CW-pRIS was confirmed by in vivo fluorescent imaging as described above 3 days after the application of DNV topical formulation. Specifically, 20 h after ZOL-IV injection, the mice were randomly divided into three groups (*n* = 8 per group, *n* = 24 in total) for topical treatment with 800CW-pRIS-DNV solution once or twice a week, as well as topical treatment with MiliQ water as a negative control. The mice were anesthetized by isoflurane inhalation and 3 µl of 100 µM 800CW-pRIS-DNV was applied on the palatal gingival tissue for each time application in the 800CW-pRIS-DNV treated groups. After the topical application of 800CW-pRIS-DNV or MiliQ water, the maxillary left first molar was extracted for each mouse as above. Fourteen days after the tooth extraction, mice were euthanized and the maxillary tissues were harvested for intra-oral photographs, micro-CT imaging and histological preparation for the assessments as described below.

### Outcome assessments of tooth extraction wound healing

In this study, all mice received a ZOL IV injection followed by tooth extraction. The experimental groups were further treated with 800CW-pRIS by intra-oral injection or as a formulation in DNV applied topically. The outcome for each experimental group was compared to the appropriate control groups.

#### Gingival swelling (Fig. S7)

Using the intra-oral photographs, the swelling area of oral mucosa around the tooth extraction socket was measured by ImageJ (NIH, Bethesda, MD) and normalized by normal mucosa surrounding the maxillary right first molar.

#### Micro CT analysis (Fig. S8)

The extracted maxilla was fixed in neutral buffered formalin. The fixed maxilla was scanned with micro-CT machine (SkyScan 1172 scanner, Skyscan, Kontich, Belgium) at source voltage of 60 kV and source current of 166 µA with the scanning resolution of 10 µm per pixel. New bone formation in the socket was analyzed by CTAn version 1.11 using the following protocol:

Step 1: A horizontal image of micro-CT containing the extraction sockets and the corresponding contralateral remaining mesial root, palatal root and distal root were identified.

Step 2: The cross-sectional area of the remaining mesial root, palatal root and distal root was determined.

Step 3: The mirror image of the cross-sectional area of remaining roots was transferred to the tooth extraction side, identifying the “original” tooth extraction socket area.

Step 4: A total of 35 layers covering from the apex of the identified tooth extraction cross-sectional area were stacked up. The bone volume over tissue volume (BV/TV) in each extraction socket was determined and combined.

#### Histological osteonecrosis analysis

After scanning the maxilla with micro-CT, the maxillary samples were fixed with 70% ethanol and decalcified with 10% EDTA in tris buffer. The conventional paraffin-embedded cross-sections were made through the medial root (mesial section) or the palatal/distal root (distal section) of the remaining right first molar and stained with hematoxylin and eosin. The sections containing palatal bone of the middle and distal area of tooth extraction site were used for measuring the osteonecrosis area. The osteonecrotic bone area, which was defined as the presence of a cluster of four or more empty osteocytic lacunae, was measured by ImageJ (NIH, Bethesda, MD) and standardized by the occlusal half cross-section area of the palatal bone.

#### Histological inflammation index analysis (Fig. S9)

All sections with H-E staining were reviewed and assessed with visual inflammatory index system (0 = normal, 1 = mild, 2 = moderate, 3 = severe) by three blinded investigators.

Inflammation Index 0: No inflammatory cell infiltration in the palatal gingiva

Inflammation Index 1: Mild inflammatory cell infiltration, localized on the maxillary bone surface.

Inflammation Index 2: Moderate inflammatory cell infiltration occupying <50% area of the palatal gingiva

Inflammation Index 3: Severe inflammatory cell infiltration occupying more than 50% area of the palatal gingiva.

### Immunohistochemistry of Th17 and Th1 cells in the BRONJ lesion

EDTA-decalcified paraffin-embedded histological sections from the control mice and 800CW-pRIS-DNV treated mice were used for immunohistochemistry. The histological cross-sections were first subjected to the antigen retrieval process using microwave irradiation. The sections were incubated with rabbit recombinant anti-RORγt antibody or rabbit recombinant anti-T-bet antibody (EPR20006 and EPR9302, respectively, Abcam, Waltham, MA) at 1/50 dilution followed by anti-rabbit secondary antibody incubation and diaminobenzidine substrate treatment. The sections were counter stained with hematoxylin.

### 800CW fluorescent signal in mouse femurs

Two days after the intra-oral injection of 100 µM 800CW-pRIS in 2 µl saline solution or after topical application of 100 µM 800CW-pRIS-DNV in 3 µl MQW, mouse femurs were harvested and the 800CW fluorescent signal was examined by the in vivo imaging system. The region of interest was selected at the femur head (*n* = 3 per group). For the imaging control, one mouse received 100 µM 800CW-pRIS IV in 100 µl saline solution via the retro-orbital plexus.

### Statistics and reproducibility

For the statistical analysis, Student’s *t* test or one-way analysis of variance (ANOVA) with Dunnett post hoc test was used for comparisons of in vitro and in vivo data. A significant difference was defined by *p* < 0.05. Figures of numerical data contain the raw measurements, means and standard deviations. The data were showed as mean and standard error of the mean.

### Reporting summary

Further information on research design is available in the Nature Research Reporting Summary linked to this article.

## Results

### Synthesis of 800CW-pRIS

The synthesis of 800CW-pRIS is outlined in Fig. 1d. Briefly, *Para*-pyridyl-risedronate (pRIS) **3** was synthesized from 4-pyridylacetic acid and reacted with ‘Magic Linker’ (*N*-*t*Bu 3-aminopropene oxide, **2**) to give the pyridinium derivative **4** in quantitative yield^35,36^. After deprotection to **5** with HCl, reaction with IRDye 800CW **7** in activated form (as the NHS carboxyl ester, **6**) gave the desired conjugate 800CW-pRIS in 46% yield. The modification of pRIS by linking to the fluorescent 800CW dye provided a convenient means to monitor distribution of the compound in bone tissue in situ via fluorescent imaging while further suppressing any remaining antiresorptive activity^14^. The detailed synthesis procedures are found in the “Methods” section.

**Fig. 2:**
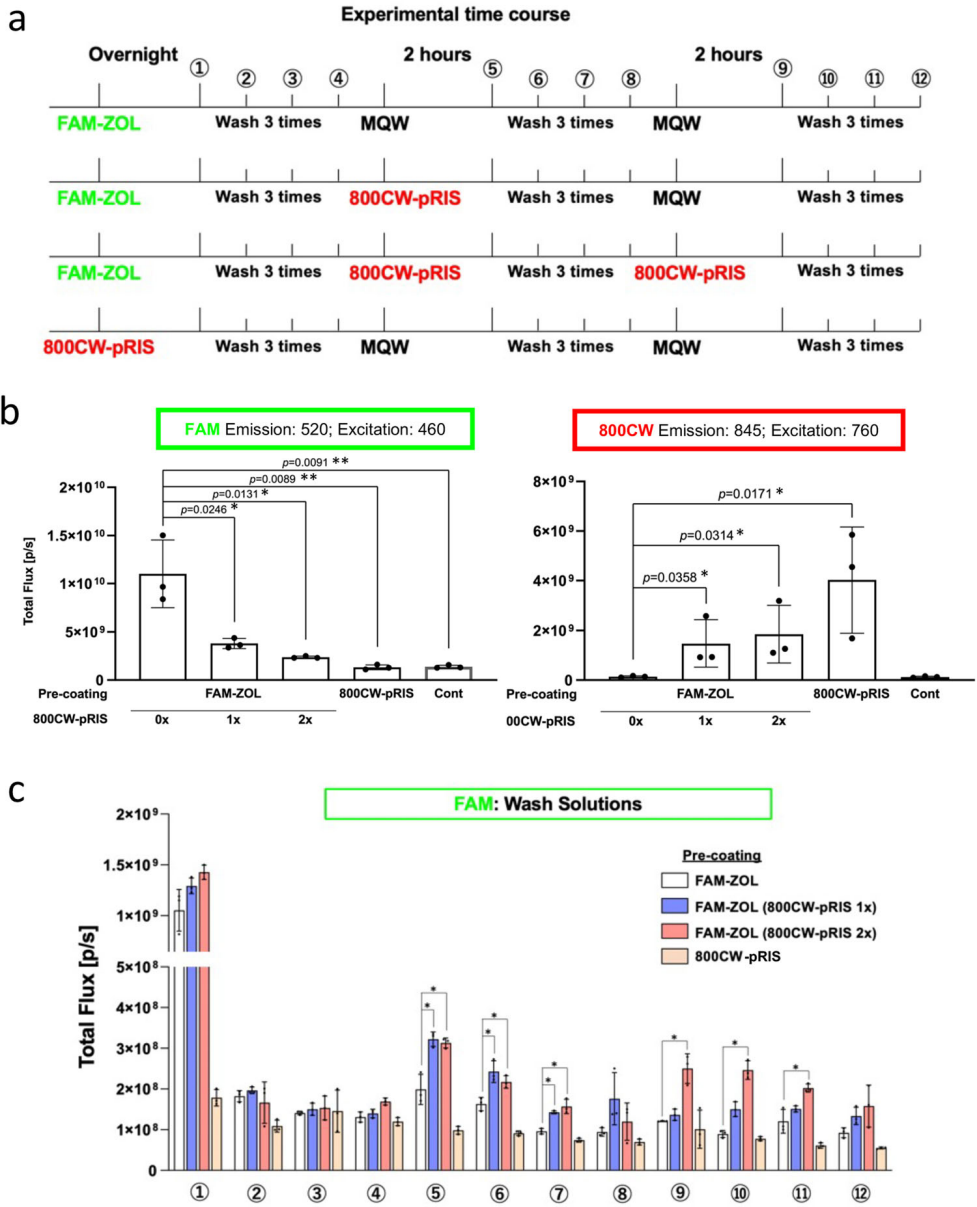
Competitive equilibrium-based dissociation of ZOL by 800CW-pRIS in vitro. **a** Experimental plan to examine the competitive equilibrium-based dissociation of N-BP (5-FAM-ZOL) by 800CW-pRIS. 5-FAM-ZOL (10 µM) was pre-adsorbed to synthetic apatite wells, which were challenged by 800CW-pRIS (10 µM in MilliQ filtered pure water: MQW) one or two times. For control application (Cont), the vehicle (MQW) was applied. **b** Fluorescent FAM and 800CW signals of the synthetic apatite wells. FAM signal was decreased after 800CW-pRIS applications, while 800CW signal was increased. **c** FAM signal of the recovered solutions after each treatment. FAM signal was increased after 800CW-pRIS application, suggesting the removal of 5-FAM-ZOL from the synthetic apatite well. The in vitro experiment was performed in triplicated wells per group (**b**, **c**). The graphs present the mean and standard deviation. Student’s *t* test was used to compare the 5-FAM-ZOL alone group and all other groups (**b**) or the 5-FAM-ZOL along group and all other groups within the indicated time points (**c**) (*n* = 3 for each group set). *p < 0.05; **p < 0.01. Source data: Supplementary Data 1, Fig. 2b, c.

To determine the postulated competitive equilibrium-based replacement efficiency of 800CW-pRIS, we performed an in vitro assay in which fluorescent-tagged 5-FAM-ZOL pre-adsorbed to the synthetic apatite wells and was challenged by once or twice 800CW-pRIS application (Fig. 2a). After 800CW-pRIS applications, the FAM fluorescent signal was significantly decreased from the well, while the 800CW fluorescent signal was increased (Fig. 2b). The collected wash solutions after the 800CW-pRIS application detected the FAM signal suggesting the removal of 5-FAM-ZOL from the synthetic apatite well (Fig. 2c). It was noted that the application of 800CW-pRIS did not completely remove 5-FAM-ZOL, however significantly lowered the concentration of 5-FAM-ZOL on the synthetic apatite well.

The displacement efficacy is dictated by the relative affinity of the two bisphosphonates for HAp binding. In a previous study we found that 800CW-ZOL has a similar binding affinity for HAp to RIS and ZOL (~0.95 for 800CW-ZOL, 1 for RIS and 1.2 for ZOL)^36^. The similar binding affinity and the great local concentration of 800CW-pRIS should mainly displace ZOL and shift the binding-equilibrium towards the dye-BP conjugate. Finally, the anti-prenylation activity and cytotoxic properties of 800CW-BP conjugates have been assessed in previous studies and shown to be negligible^36^.

### Modeling of 800CW-pRIS into the IPP site of FPPS

The observed differences in potency between early alkyl N-BPs such as pamidronate and later generation heterocyclic N-BPs such as RIS and ZOL has prompted structure-activity relationship investigations to identify specific interactions of these drugs within the FPPS active site^37,38^. It has been proposed that N-BPs inhibit FPPS by mimicking a carbocation intermediate of the allylic diphosphate, dimethylallyl pyrophosphate (DMAPP)^37^. Within the FPPS active site (PDB 1YV5), three residues, the carbonyl oxygen of Lys200 and the side chain oxygens of Thr201 and Gln239, form a carbocation-stabilizing triad by directing their respective negative dipole ends towards the carbocation of DMAPP^37^. N-BPs bind strongly to this “allylic substrate binding pocket” by coordination of their phosphonate groups via structural Mg^2+^ ions^38^. The more potent N-BPs such as RIS and ZOL strengthen their active site binding through hydrogen bonding interactions with the protonated nitrogen atom within the heterocycle, the main chain carbonyl oxygen of Lys200 and the side chain oxygen of Thr201^38^. Mutagenesis studies investigating the role of Thr201 in the binding of RIS have reported somewhat contradictory results. A conservative T201S mutation resulted in significantly increased inhibition of FPPS, however, a T201A mutation resulted in very minor change in inhibition. These mutagenesis experiments confirm that Thr201 does form binding interactions with N-BP inhibitors, however, the interaction might not be essential for inhibition of FPPS. In vitro studies have attempted to relate this capability of hydrogen bonding with Thr201 to inhibition of the enzyme. The in vitro results showed a significant reduction in IC_50_ values for the inhibition of FPPS when comparing RIS and its *ortho*-pyridyl analogue (oRIS), 5.7 ± 0.54 nM and 9.2 ± 0.96 nM respectively^39^. The reduced inhibition of oRIS compared to RIS encouraged us to explore pRIS as a conjugation partner to the 800CW dye, wherein the pyridyl nitrogen is more favorably located for conjugation but less able to interact with the FPPS binding site.

**Fig. 3:**
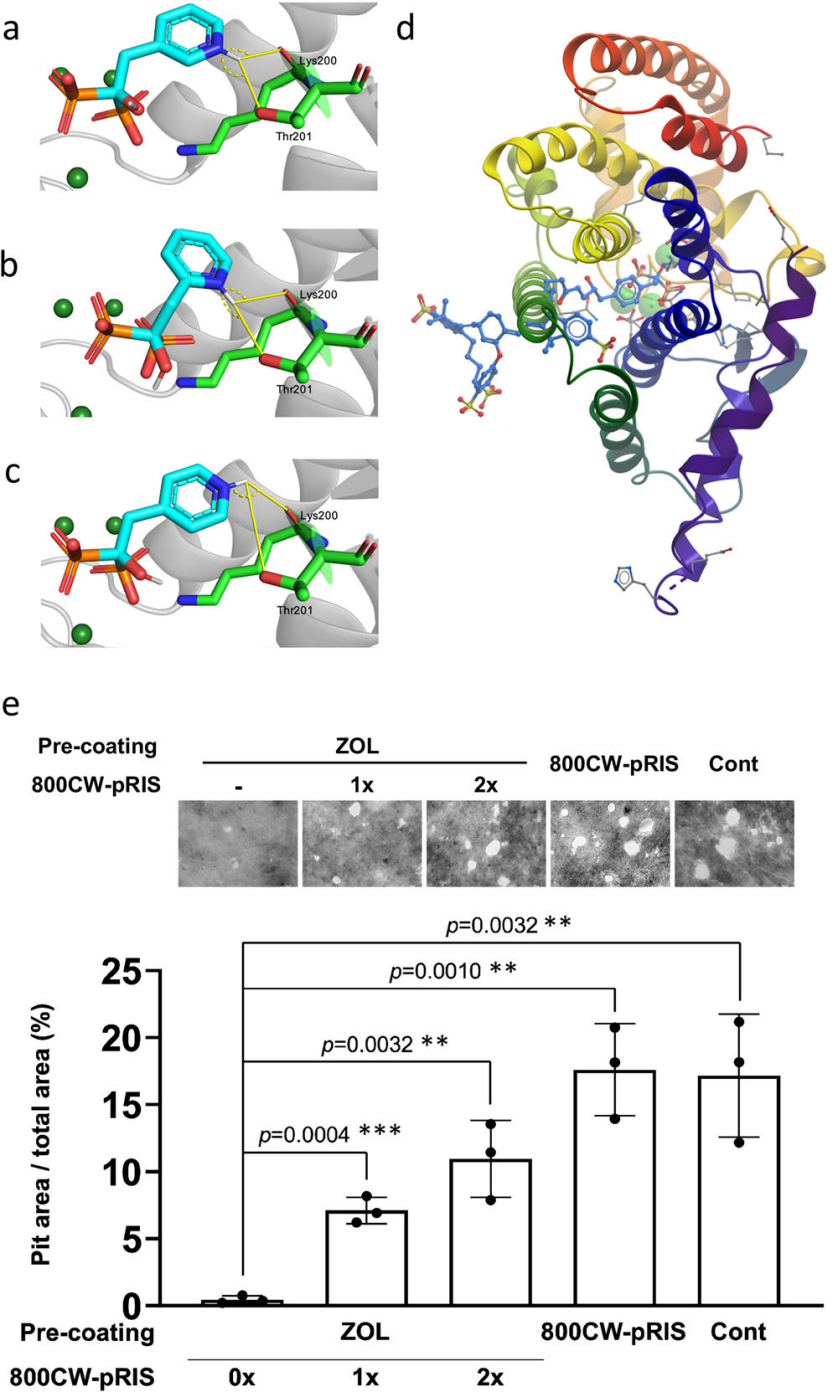
800CW-pRIS docking studies of N-BPs binding human FPPS (PDB 1YV5) in silico and its effect on osteoclastic resorption pit formation in vivo. **a** *meta*-risedronate (RIS) docked into active site. **b** *ortho*-RIS docked into active site. **c** *para*-RIS docked into active site. Figures were generated using PyMol. Code: yellow line—hydrogen bond; dashed yellow line—hydrogen bond angles; cyan structures—N-BP carbon backbone; blue atoms— nitrogen; red atoms—oxygen; green spheres—Mg^2+^; green structure— Lys200 and Thr201 carbon backbone. **d** 800CW-pRIS built from within the relevant FPPS active site (PDB 1YV5). Figure was generated using ICM-Pro 3.2. Code: blue structure—800CW-pRIS carbon backbone; red atoms— oxygen; yellow atoms—sulfur; green spheres—Mg^2+^. **e** Functional characterization of 800CW-pRIS as a low potency BP using the in vitro pit formation assay by mouse osteoclasts. Synthetic apatite coated wells were treated with 10 µM ZOL, 10 µM 800CW-pRIS or MilliQ water followed by washes. Some ZOL-treated wells were further treated with 10 µM 800CW-pRIS one time or two times and washed. Mouse osteoclasts derived from RAW274.1 cells were inoculated, and the total area of resorption pits were measured. The in vitro experiment was performed in triplicated wells per group. The graphs present the mean and standard deviation. Student’s *t* test was used to compare the ZOL alone group and all other groups (*n* = 3 for each group set). **p < 0.01; ***p < 0.001. Source data: Supplementary Data 1, Fig. 3e.

In silico docking experiments were performed using the AutoDock Vina software and ligands were prepared using AutoDockTools 1.5.6^32^. The crystal structure of human FPPS (PDB 1YV5) was used. We show the docking mode of each RIS analogue that best corresponds to that of bound RIS in the FPPS active site. To verify the validity of our docking method, we compared the root-mean-square deviation (RMSD) between the docked RIS ligand with the crystallographic RIS pose. We calculated a RMSD of 1.67 Å which is below the commonly used RMSD ≤ 2 cutoff^33^. This verifies that our ligand was correctly posed in the target active site. With the pyridyl nitrogen in the *meta* position (Fig. 3a), our modeling calculates a binding energy of −8.1 kcal/mol. We measured a hydrogen bonding distance between the hydrogen and the T201 oxygen of 2.4 Å and an O-H-N angle of 138.6°, and a hydrogen bonding distance between the hydrogen and the K200 carbonyl oxygen of 2.5 Å with an O-H-N angle of 115.2°. Moving the nitrogen to the *ortho* position (Fig. 3b), we expect to see moderately decreased binding affinity of the ligand due to disruption of the bifurcated hydrogen bonding. Indeed, the calculated binding energy was decreased slightly to −7.3 kcal/mol, which is consistent with a higher IC_50_ value. In this binding mode we measured a hydrogen bonding distance of 2.7 Å and an angle of 158.7° between the nitrogen and the T201 oxygen, with a hydrogen bonding distance of 2.3 Å and an angle of 104.4° between the hydrogen and the K200 carbonyl oxygen. The final docking of pRIS (Fig. 3c) resulted in a comparable binding energy to oRIS of −7.4 kcal/mol and a hydrogen bonding distance of 4.2 Å with an angle of 78.8° between the hydrogen and the T201 oxygen and a hydrogen bonding distance of 2.2 Å with an angle of 128.5° between the hydrogen and the K200 carbonyl oxygen.

These results are consistent with previous studies on the RIS and oRIS co-crystal structures and suggested that pRIS should be a weaker inhibitor of FPPS than RIS^38^. However, as the in vitro results demonstrate, the corresponding reduction in IC_50_ for these RIS analogues is likely not quite sufficient to qualify them as pharmacologically inactive. Based on our prior work on N-BP conjugates with a far-red fluorescent dye, AF647^14^, conjugation of pRIS with the yet bulkier infrared dye 800CW was envisaged to provide a potential in vivo imaging tag on the N-BP while eliminating any remaining pharmacological effect on osteoclasts^14^. Attempts to dock 800CW-pRIS to the FPPS active site resulted in weak, non-specific binding on the exterior surface of the enzyme (Fig. S10). To force this bulky N-BP conjugate into the relevant FPPS active site, we attempted to build the molecule from within the site using ICM-Pro 3.2. During the building process the structure was progressively minimized to ensure optimal conformations. The resulting binding mode of 800CW-pRIS, when forced to stay within the active site, is shown in Fig. 3d. It is evident that significant steric clashes with multiple residues preclude active site binding, indicating that 800CW-pRIS should be pharmacologically inactive and thus a good candidate as a benign displacement N-BP with the added virtue of providing an inherent imaging fluorophore to localize the compound when adsorbed on bone mineral surfaces.

To validate the lack of pharmacological activity of 800CW-pRIS predicted by the in silico modeling, we performed an in vitro study to test the effect of 800CW-pRIS on osteoclastic pit formation. The synthetic apatite-coated culture wells were treated with 10 µM ZOL or 10 µM 800CW-pRIS. Mouse osteoclasts derived from RAW264.7 cells were inoculated to each well and the generated pit area was measured. ZOL-treated wells significantly decreased the pit area suggesting that osteoclastic activity was pharmacologically inhibited, whereas 800CW-pRIS-treated synthetic apatite wells maintained the active osteoclastic activity at the level of negative control wells (Fig. 3e). Furthermore, ZOL-pretreated wells were challenged by 800CW-pRIS as shown in Fig. 2a. The larger osteoclastic pits were formed in 800CW-pRIS challenged wells, suggesting that the competitive equilibrium-based removal of ZOL attenuated the downregulation of osteoclastic activity (Fig. 3e). Taken together, our data strongly supported that the newly designed 800CW-pRIS lacked the pharmacological activity, while maintained the affinity to bone mineral.

### Intra-oral injection of 800CW-pRIS prior to tooth extraction in ZOL-injected mice

**Fig. 4:**
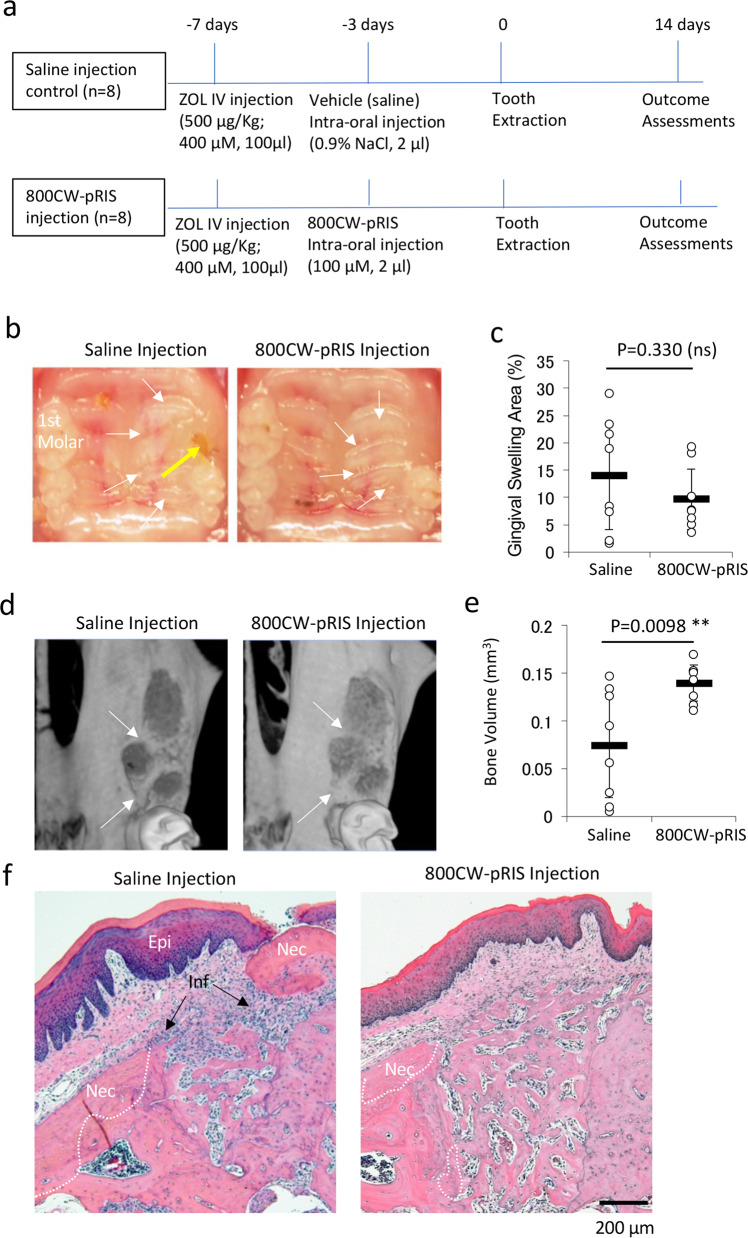
The effect of 800CW-pRIS intra-oral injection on tooth extraction wound healing in mice pretreated with ZOL. **a** Experimental protocol. **b** Intra-oral photographs of mouse maxilla 2 weeks after tooth extraction depict the gingival swelling area (white arrows) and open wound (yellow arrow). **c** Gingival swelling area standardized by the circumferential area of the right maxillary first molar (*n* = 8 per group). **d** Three-dimensional reconstruction of maxillary bone containing extraction sockets. The bony socket edge (while arrows) was well maintained in the saline injection group, while underwent bone resorption in the 800CW-pRIS injection group. **e** Micro-CT measurement of bone volume in the extraction sockets (*n* = 8 per group). **f** A representative histological cross-section of the saline injection group showed osteonecrosis (Nec) and gingival inflammation (Inf). It was noted that a piece of necrotic bone sequestration exposed to the oral cavity through hyperplastic epithelium (Epi). A representative histological cross-section of the 800CW-pRIS injection group showed active regenerating bone in the socket with less extensive gingival inflammation and osteonecrosis. The graphs present the mean and standard deviation. Student’s *t* test was used. **p < 0.01. Source data: Supplementary Data 1, Fig. 4c, e.

We have established a mouse model involving a bolus intravenous (IV) injection of 400 µM ZOL followed by maxillary first molar extraction^21,22^. In the present study, mice received a 100 µM 800CW-pRIS injection to the left palatal gingival tissue after the ZOL IV injection following the previously published protocol^14^. Three days after the intra-oral injection, the left maxillary first molar was extracted. Two weeks after the tooth extraction, mouse maxillary tissue was harvested for outcome assessment (Fig. 4a).

ZOL-pretreated control mice receiving a saline intra-oral injection showed delayed tooth extraction wound healing. Gross anatomical observation revealed that the gingival wound healing of mice treated with ZOL developed abnormal appearance with ischemic swelling, which appeared to be collectively contributed by decreased vasculature structures, epithelial hyperplasia, and gingival inflammation (Fig. S7). The palatal gingiva in mice receiving the 800CW-pRIS intra-oral injection appeared to be better than that of saline injected, ZOL-pretreated mice (Fig. 4b), however the reduction of the gingival swelling area was not statistically significant (Fig. 4c). Three-dimensional micro-CT images showed that the tooth extraction sockets of the mesial, palatal, and buccal roots of the maxillary first molar exhibited no surgical complications. Tooth extraction induces a distinct bone resorption on the external surface of residual alveolar bone modulating the socket edge^40^. The ZOL-pretreatment appeared to have preserved the tooth socket morphology in the saline injection group, while the 800CW-pRIS injection group showed the sign of osteoclastic bone resorption at the socket edge of residual alveolar bone (Fig. 4d).

The bone volume over total volume within the extraction sockets was determined using micro-CT images (Fig. S8). The combined BV/TV of all the three extraction sockets of saline-injected disease control mice varied among individual animals, whereas the extraction socket of 800CW-pRIS injected mice was uniformly filled by regenerating bone (Fig. 4e). Histological cross-sections of the extraction socket corroborated the new bone formation in 800CW-pRIS injected mice, with less inflammatory cell infiltration in the overlining gingival tissue. Tooth extraction induces the bone regeneration from the bottom of the bony socket, which was observed in both saline and 800CW-pRIS injection groups. However, the extensive inflammatory reaction in the saline injection group might have delayed the completion of extraction socket healing. Furthermore, a piece of necrotic bone appeared to be sequestered and exposed through hyperplastic oral epithelium of a saline-injected mouse (Fig. 4f).

**Fig. 5:**
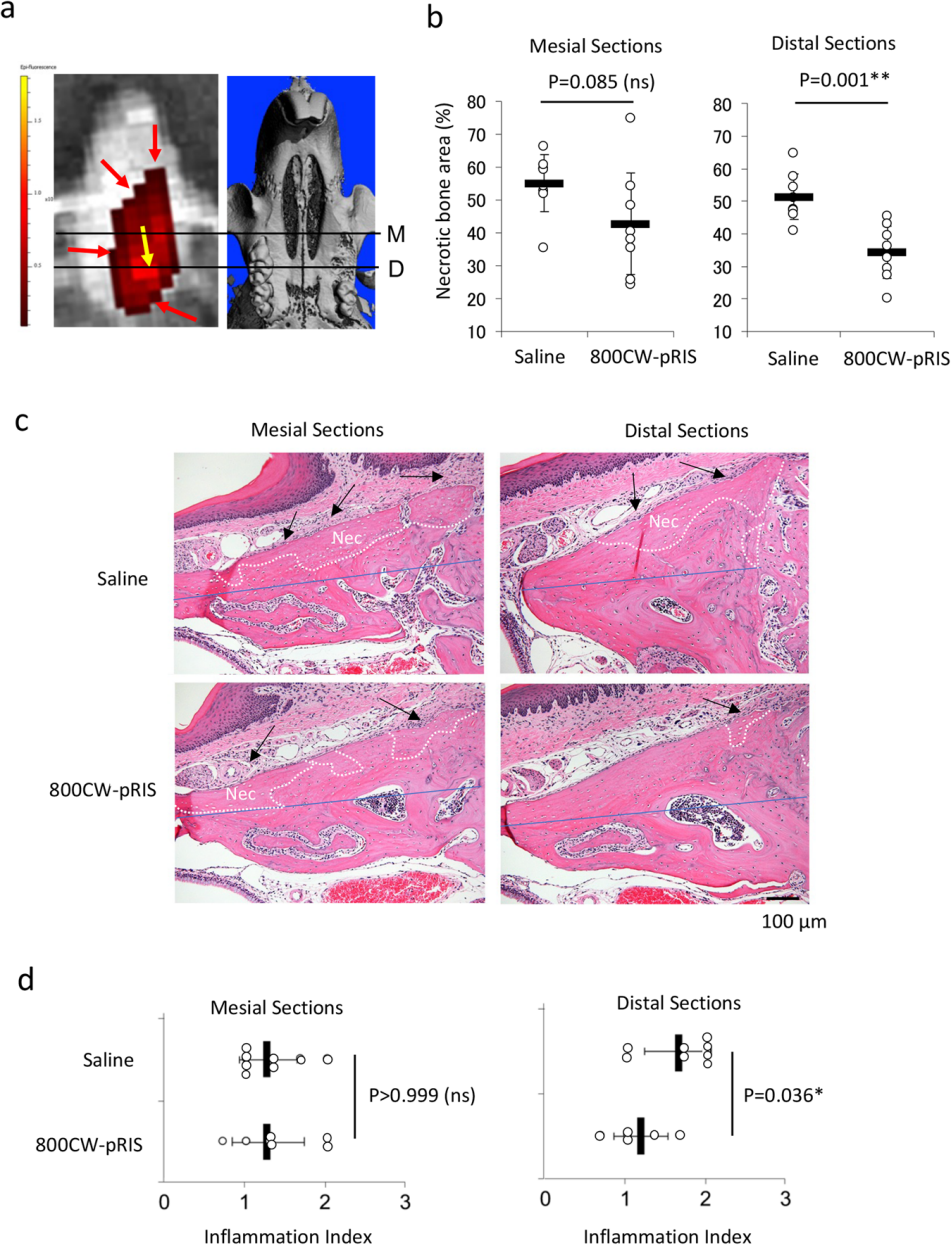
The effect of 800CW-pRIS intra-oral injection on osteonecrosis and gingival inflammation around socket in mice pretreated with ZOL. **a** Distribution of 800CW-pRIS after intra-oral injection examined by IVIS and the corresponding micro-CT image. An intense 800CW signal is observed in the direction of injectant (yellow arrow) from the injection site and a weaker signal is seen in the peripheral zone (red arrows). The mesial section (M) has relatively less signal intensity compared to the distal section (D). **b** Percent area of osteonecrosis over the occlusal half of maxillary alveolar bone measured in the medial and distal sections (*n* = 8 per group). **c** Histological cross-sections of mesial and distal tooth extraction area with osteonecrosis (white dotted line: Nec) and inflammation (Inf and black arrows). The osteonecrosis area was normalized by the occlusal half of maxillary bone indicated by blue line. **d** Inflammation index (0–3) was evaluated by three blinded examiners. The graphs present the mean and standard deviation (*n* = 8 per group). Student’s *t* test was used. *p < 0.05; **p < 0.01. Source data: Supplementary Data 1, Fig. 5b, d.

IVIS observation revealed that strong 800CW NIR signal on the palatal bone; however, 800CW signal was not uniform among animals. 800CW NIR signal was prominent along the vector of the injectant toward the distal area, while the surrounding area demonstrated a weaker 800CW NIR signal (Fig. 5a). The effect of 800CW-pRIS injection was evaluated in two separate areas: cross-sections through (1) the mesial root (mesial sections) and (2) through the palatal-distal roots (distal sections). The osteonecrosis area was observed to be smaller in 800CW-pRIS injected mice than in saline-injected control mice; however, statistical significance was only attained in the distal sections (Fig. 5b). Histological evaluation also revealed inflammatory cell infiltration in the gingival tissue. The extent of inflammation was visually determined by three blinded researchers reporting the inflammation index with scores of 0 to 3: 3 being the most significant inflammation (Fig. S9). It was found that the inflammation index was decreased only in the distal sections of 800CW-pRIS injected mice (Fig. 5d).

### 800CW-pRIS in DNV topical formulation

**Fig. 6:**
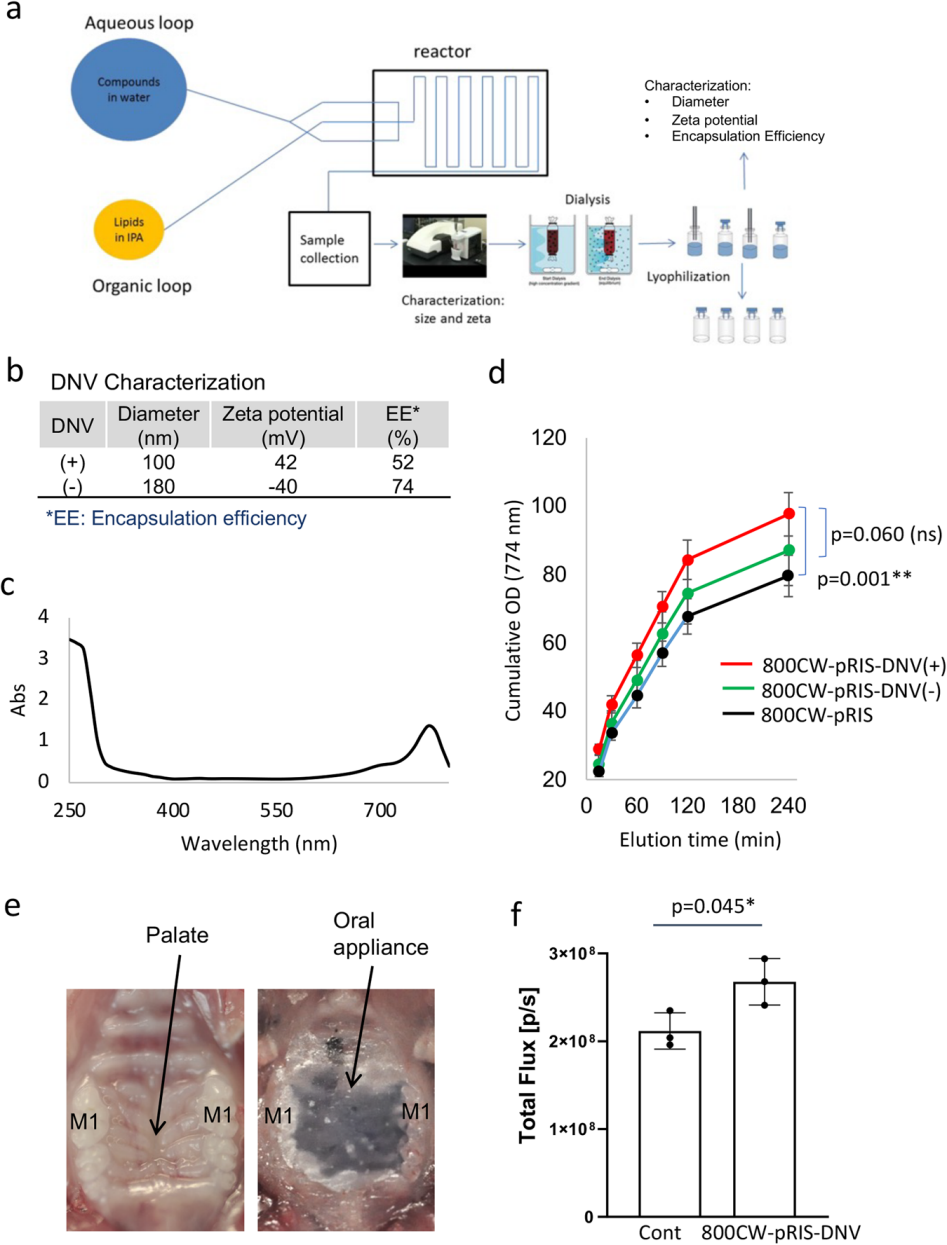
Synthesis of 800CW-pRIS-DNV and characterization of trans-oral mucosa delivery in vitro and in vivo. **a** Diagram of micro-fluidics DNV synthesis. **b** Size and surface charge of DNV containing 800CW-pRIS with the encapsulation efficiency (EE). **c** Absorption spectrum of 800CW-pRIS-DNV. **d** Time course detection of 800CW-pRIS-DNV after elution through human gingival epithelial culture. The in vitro experiment was performed in 12 wells per group. The assay medium was changed every 30 min and measured the absorbance in a spectrophotometer at 774 nm. The optical density (OD) measurement per proup was described as the cumulative OD data. One-way analysis of variance (ANOVA) with Tukey post hoc test was used per time point. **e** Protocol for topical application of 800CW-pRIS-DNV to mouse palatal tissue demonstrated on mouse cadaver maxilla. A custom-made oral appliance covered the palatal tissue to retain the 800CW-pRIS-DNV formulation. **f** 800CW fluorescent signal detected in mouse maxillary tissue 3 days after topical application of 800CW-pRIS-DNV (*n* = 3 per group). The graphs present the mean and standard deviation. Student’s *t* test was used. *p < 0.05. Source data: Supplementary Data 1, Fig. 6d, f.

These results prompted us to seek a more uniform administration than provided by the direct intra-oral injection: specifically, a method for topical formulation of 800CW-pRIS. We chose microfluidics-synthesized deformable nanoscale vesicle (DNV) as the delivery agent (Fig. 6a). DNV is a liposome derivative that has been demonstrated to effect trans-epithelial N-BP delivery through mouse skin tissue^34^. The delivery performance of liposome derivatives is affected by their size and surface charge^41^. In this study, we set the DNV size within a range of 100~200 nm and evaluated the effect of surface charge on passage through the gap junction of the oral epithelial layer (Fig. 6b). 800CW NIR signal (Fig. 6c) was used to detect elution of 800CW-pRIS-DNV through a commercially available in vitro human gingival epithelial layer model. Positively charged 800CW-pRIS-DNV showed the best permeation activity throughout the elution period (Fig. 6d).

This in vitro study further suggested that the effect of DNV-derived trans-oral epithelial drug delivery should be cumulative over time. Therefore, we developed an in vivo application protocol to sustain the trans-epithelial delivery to mouse palatal tissue for 1 h. To retain the topically applied 800CW-pRIS-DNV formulation, an oral appliance was custom-made using dental resin (Fig. 6e). Mouse maxillary bone generated a measurable NIR signal 3 days after the topical application of 800CW-pRIS-DNV (Fig. 6f) that confirmed successful delivery of 800CW-pRIS to the maxillary jawbone in a single application.

### 800CW-pRIS-DNV applied to palatal tissue attenuated the delayed tooth extraction wound healing in ZOL-pretreated mice

**Fig. 7:**
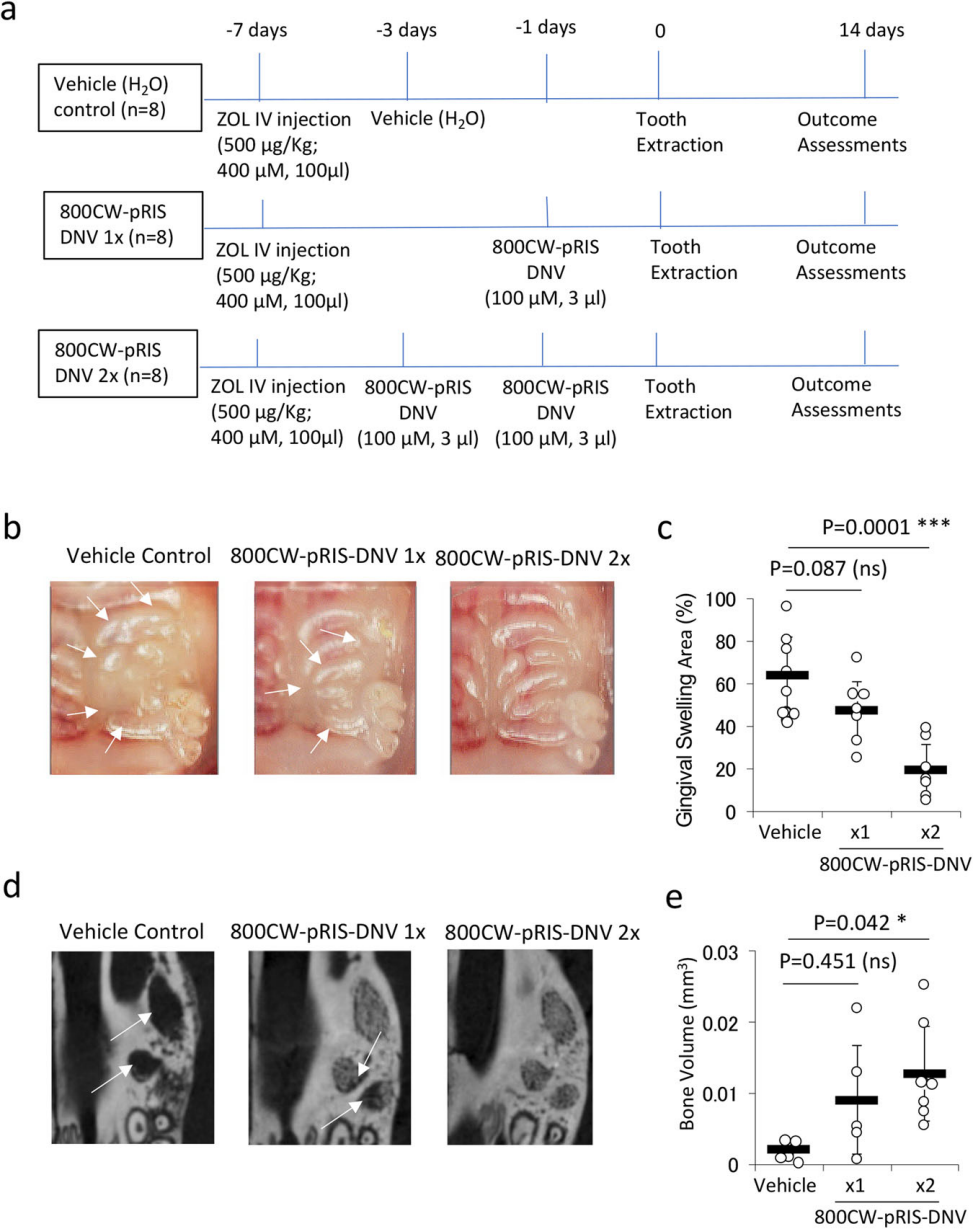
Effect of 800CW-pRIS-DNV applied topically to mouse palatal tissue on tooth extraction wound healing of mice pretreated with ZOL. **a** Experimental protocol. **b** Intra-oral photographs depict gingival swelling (white dotted line) and the open tooth extraction wound (yellow arrows). **c** The gingival swelling area was dose dependently decreased by topical application of 800CW-pRIS-DNV (*n* = 8 per group). **d** Three-dimensional reconstruction of micro-CT images shows a region of osteoclastic resorption pits at the tooth extraction site (white arrow) after two applications of 800CW-pRIS-DNV. **e** The bone volume in the tooth extraction sockets dose-dependently increased with topical application of 800CW-pRIS-DNV. Graphs present mean and standard deviation (*n* = 5~7 per group). One-way analysis of variance (ANOVA) with Dunnett post hoc test was used. *p < 0.05; ***p < 0.001. Source data: Supplementary Data 1, Fig. 7c, e.

We developed a treatment protocol with repeated topical applications of 800CW-pRIS-DNV prior to the tooth extraction. After a bolus ZOL IV injection, 800CW-pRIS-DNV reconstituted in pure water was topically applied to the mouse palatal gingiva for 1 h. A group of ZOL-pretreated mice received twice repeated 800CW-pRIS-DNV topical applications. All mice underwent tooth extraction (Fig. 7a). Two weeks after tooth extraction, the gingival wound appeared to be better healed in the mice receiving twice repeated topical applications of 800CW-pRIS-DNV than the vehicle-only controls (Fig. 7b). Gingival swelling defined as the area of gingival tissue with less prominent palatal rugae structure was markedly reduced in mice receiving the twice-repeated topical treatments with 800CW-pRIS-DNV (Fig. 7c). Micro-CT images of the extraction sockets showed bone regeneration by 800CW-pRIS-DNV treatment (Fig. 7d). The bone volume measured within the extraction socket was increased in the twice-repeated 800CW-pRIS-DNV treated group with statistical significance (*p* = 0.042) (Fig. 7e). In vehicle control and once topical application of 800CW-pRIS-DNV group, two mice at each group were excluded from micro CT analysis because of root remaining.

**Fig. 8:**
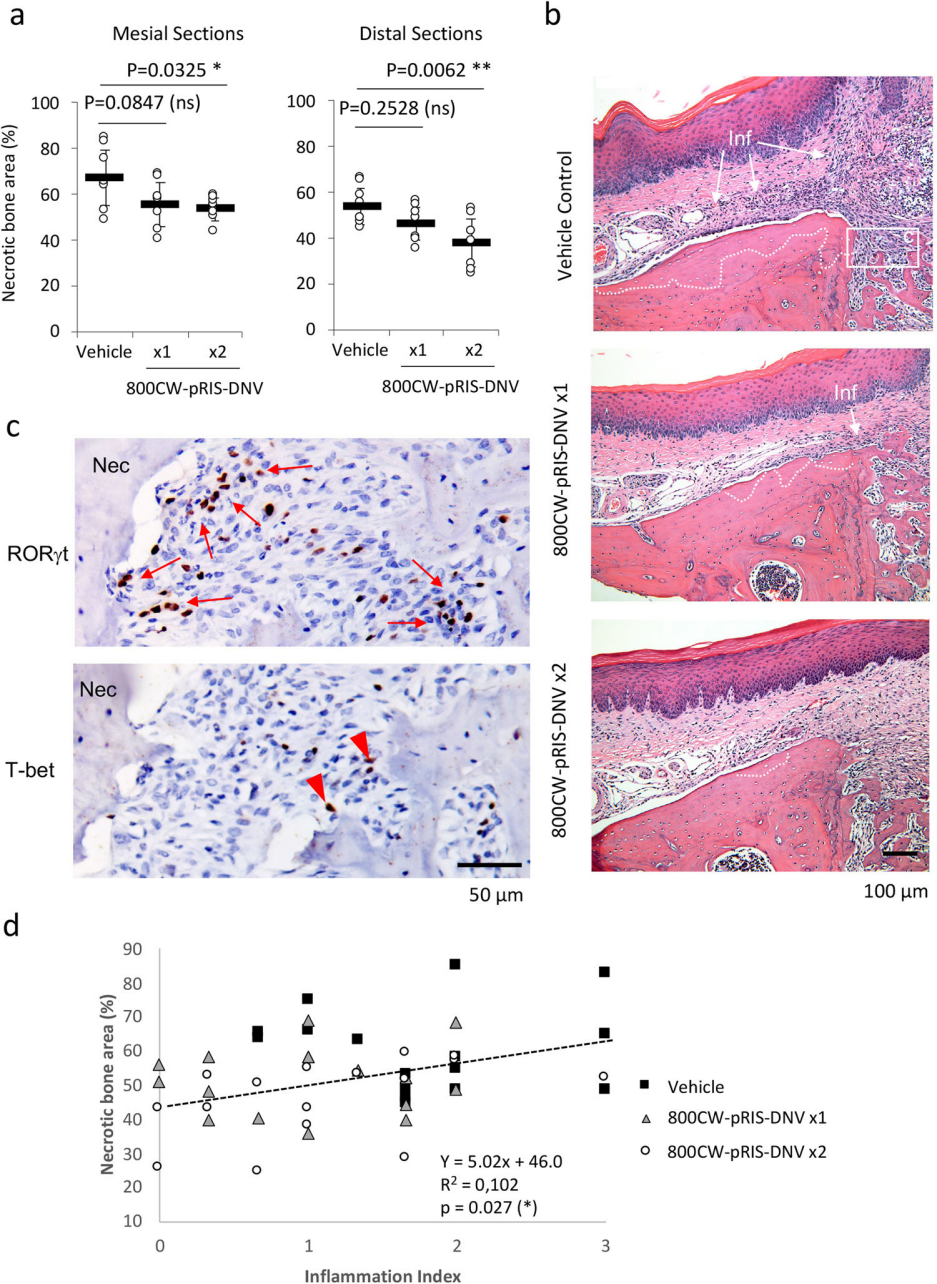
Histological characterization of tooth extraction wound with 800CW-pRIS-DNV treatment in mice pretreated with ZOL. **a** The area of osteonecrosis is dose-dependently decreased by topical application of 800CW-pRIS-DNV. The mesial and distal sections exhibit a consistent result. Graphs present mean and standard deviation (*n* = 8 per group). One-way analysis of variance (ANOVA) with Dunnett *post hoc* test was used. *p < 0.05; **p < 0.01. **b** Representative histological cross-sections through the extraction socket of mesial root show a decreasing osteonecrosis area (white dotted line) and inflammatory cell infiltration (Inf, white arrows) after 800CW-pRIS-DNV topical treatment. **c** Immunohistochemistry of the signature transcription factors RORγt and T-bet demonstrates the presence of Th17 (red arrows) and Th1 (red arrowheads) cells, respectively in the inflammatory area of BRONJ lesion in vehicle control mice. **d** The inflammation index correlated with the osteonecrosis area (*n* = 8 animals per group; *n* = 16 data points per group reflecting mesial and distal measurements per animal). *p < 0.05. Source data: Supplementary Data 1, Fig. 8a, d.

Histological cross-sections through medial and palatal/distal roots similarly showed a decreased osteonecrosis area for the 800CW-pRIS-DNV treated mice, with the statistical significance again reached between the vehicle control and twice-repeated treatment groups (Fig. 8a). Notably, inflammatory cell infiltration had subsided in the gingival tissue at the tooth extraction site of the 800CW-pRIS-DNV treated mice (Fig. 8b). Pro-inflammatory T helper cells such as Th17 and Th1 cells have been reported in gingival inflammation^42^. The signature transcription factors, retinoid-related orphan receptor gamma (RORγt)^43^ and T-box 1 transcription factor (T-bet)^44^, respectively, were identified in the vehicle control sections (Fig. 8c) but not in 800CW-pRIS-DNV treated sections. A cluster of RORγt-positive cells were observed near the surface of necrotic alveolar bone, whereas T-bet-positive cells were sporadically observed. A comparative analysis of the combined data for osteonecrosis area and inflammation index identified a weak correlation with a statistical significance (*p* = 0.027) (Fig. 8d). Taken together, our data supported that the attenuation of gingival inflammation and osteonecrosis was achieved by twice-applied 800CW-pRIS-DNV.

### 800CW-pRIS pharmacokinetics

**Fig. 9:**
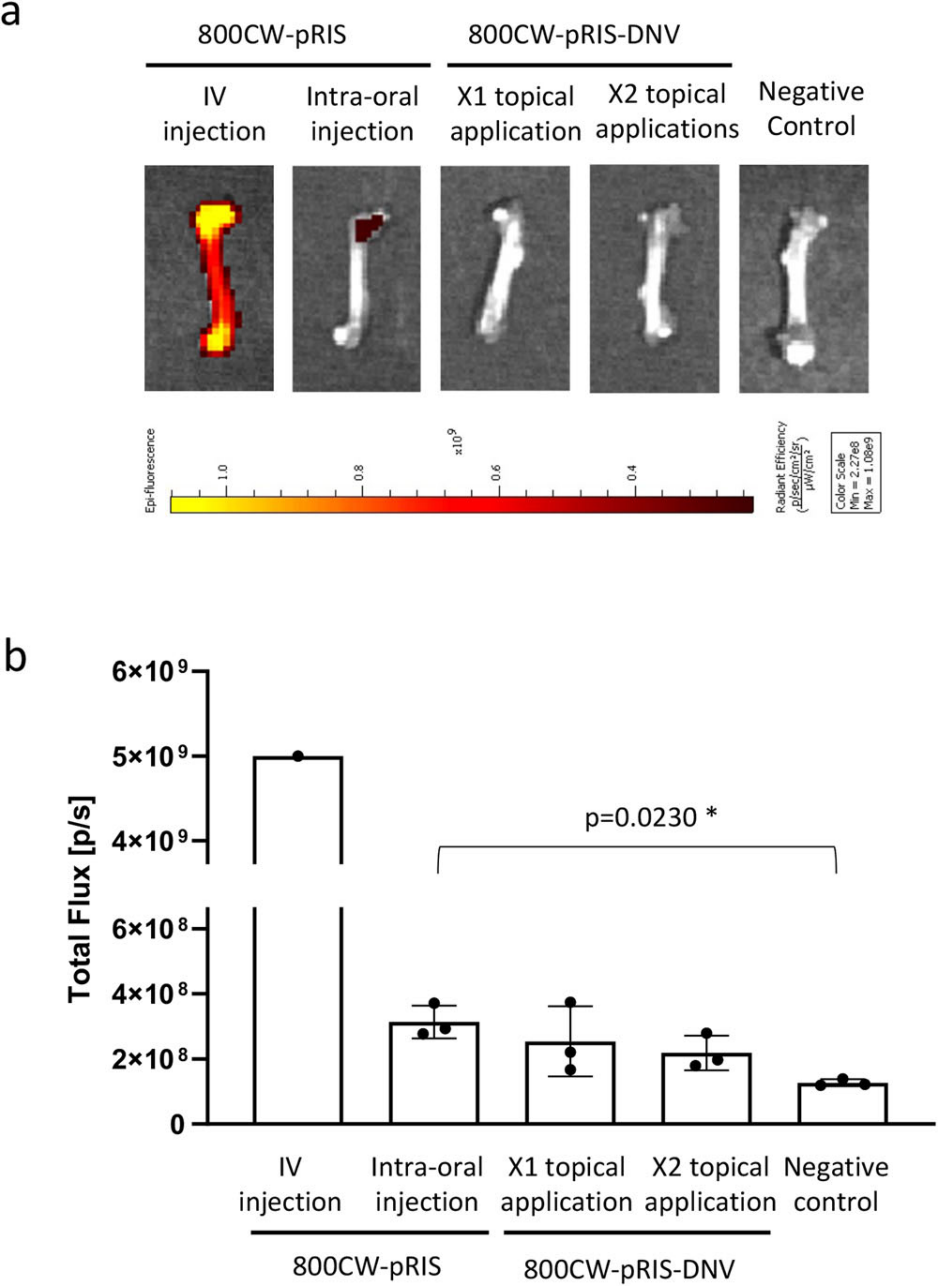
Effect of 800CW-pRIS intra-oral administration to the distant femur. **a** Mouse femurs examined by the in vivo imaging system. **b** 800CW fluorescent signal measured in the region of interest at the femur head (*n* = 3 per group; *n* = 1 for IV injection control). One-way analysis of variance (ANOVA) with Dunnett *post hoc* test was used for topical application groups. *p < 0.05. Source data: Supplementary Data 1, Fig. 9b.

To evaluate the systemic distribution of 800CW-pRIS 3 days after intra-oral application, mouse femurs were harvested and subjected to IVIS NIR evaluation (Fig. 9a). In this study, 100 µM 800CW-pRIS was injected via IV (positive control) or to the palatal mucosa. 800CW-pRIS-DNV (100 μM drug) was also applied topically either once or twice, using the protocol already described. The 800CW NIR signal in femurs harvested from mice that received the intra-oral injection of 800CW-pRIS was slightly higher than the level of negative control femurs, whereas once- or twice-applied topical applications of 800CW-pRIS-DNV did not increase the femur 800CW signal above background (Fig. 9b).

## Discussion

We have previously reported that the presence of N-BP on the jawbone surface above a likely threshold level was associated with the abnormal tooth extraction wound healing involving the development of osteonecrosis in mice^14^. We envisioned a therapeutic modality to remove pre-adsorbed jawbone N-BP locally below such a threshold level. To this end, a competitive displacement therapy was propounded using a pharmacologically inactive N-BP (Fig. 1) as the displacing agent. During the later historical development of the bisphosphonate drug class, N-BP analogues mimic binding of the natural substrate molecules DMAPP/GPP to the target enzyme, FPPS^45^. Moving the RIS *meta*-pyridyl nitrogen^46^ to the *para* position in pRIS significant decreases potency^26^. Because the chemisorption ability to HAp of pRIS is well maintained through its P-C-P bisphosphonate motif, which is structurally identical to that in RIS, we selected pRIS as an inactive N-BP for our BRONJ abatement studies. Its inhibition of osteoclast activity was further diminished by conjugation to a fluorescent dye, IRDye 800CW (Fig. 3), which provided a convenient means to monitor the location and concentration of the conjugate in situ. We previously demonstrated that conjugation of large fluorescent dyes to N-BP drugs reduced their potency in a standard in vitro prenylation assay used as a marker for antiresorptive drugs targeting FPPS^14^. IRDye 800CW is a non-toxic, next-generation near infrared fluorophore (NIR) with Ex_max_ of 778 nm and Em_max_ of 794 nm^47^. IRDye 800CW has been used as a promising clinical imaging tool in early clinical trials^48,49^. NIR fluorescence has the advantage of permitting high-resolution imaging through superficial tissue layers in molecular fluorescence guided surgery^50^. Therefore, we postulated that this fluorescent conjugate would have enhanced clinical value by combining prophylactic or therapeutic benefit with a convenient means to monitor indirectly local replacement of legacy N-BP in the treated oral cavity.

Tooth extraction is a well-recognized risk factor for the development of MRONJ^51–53^. Because tooth extraction is a terminal surgical treatment for advanced stages of dental caries and periodontal disease, it has been postulated that the preceding diseases such as periodontitis may increase the risk of MRONJ. A recent systematic review and meta-analysis of five cohort studies and seven case-control studies reported that periodontitis diagnosis was more prevalent in MRONJ patients than the control cohort^54^. However, because all clinical studies selected in this systematic review were retrospective studies in which the periodontitis evaluations were performed after MRONJ occurred, the authors discussed the limitation in data interpretation that “there was incomplete data regarding the majority of the risk factors at their onset time point^54^.”

It must be noted that the pathological mechanism of MRONJ including BRONJ has not been fully established. Animal models were investigated to address this challenge. Kim et al. investigated the role of ligature-induced periodontitis in mice treated with ZOL or anti-RNAKL antibody. MRONJ-like lesion was developed after extraction of the affected tooth^55^. This study also examined the effect of removal of ligature mimicking the treatment of periodontitis, which normalized pro-inflammatory cytokine expression. However, tooth extraction in the normalized mice also developed MRONJ-like lesion albeit with less extensive osteonecrosis area. Aguirre and his research team investigated the risk of developing BRONJ using rice rats (Oryzomys palustris) that develop naturally occurring periodontitis with high sucrose diet. They reported that preventing or controlling periodontitis of rice rats switching to normal diet reduced the occurrence of BRONJ^24^. These animal studies suggest that active periodontitis may contribute to the severity of disease phenotype but not necessarily required to cause MRONJ.

Simple tooth extraction from healthy inbred mice and rats developed abnormal wound healing with ZOL treatment, which included delayed gingival wound closure, epithelial hyperplasia and the development of jawbone osteonecrosis^18^. These oral pathological phenotypes were also reported in human MRONJ lesions. However, human MRONJ clinical diagnosis has been suggested by AAOMS to be confirmed if the lesion is not resolved over 8 weeks. Because healing time of rodent and human wounds differs significantly, it is not practical to adopt human MRONJ diagnostic criteria to rodent models and it is not clearly determined if they share the identical pathological mechanism. The present study, healthy B6 mice with ZOL IV injection were used to examine tooth extraction-induced wound healing. The data interpretation must be limited to the effect of 800CW-pRIS in removing the legacy ZOL from jawbone on the improvement of tooth extraction-associated abnormal wound healing.

The present study was designed to compare delivery of a nontoxic, fluorescently tagged 800CW-pRIS by simple intra-oral injection, (Figs. 4 and 5) or topical application in a DNV-based formulation (Figs. 7 and 8) prior to tooth extraction in ZOL-pretreated mice. Both delivery methods resulted in the attenuation of tooth extraction-related osteonecrosis. Based on the in vitro assays demonstrating the effective replacement of pre-adsorbed ZOL and nearly normalized osteoclastic activity by repeated applications of 800CW-pRIS (Figs. 2 and 3), we propose that the attenuation of abnormal tooth extraction wound healing was indeed achieved by the removal of ZOL from the mouse jawbone to decrease its bioavailable concentration below the disease-causing threshold.

In the present study, immunohistochemical evaluation revealed the presence of a cluster of Th17 cells in BRONJ-related inflammatory infiltrates, the likely source of IL-17 cytokine (Fig. 8). However, the mechanism of prolonged gingival inflammation related to N-BP has not been fully elucidated. Osteoclasts differentiated from macrophage/monocyte lineage were found to retain immune cell function characterized by the cytokine secretion profile^56^, and postulated to protect against oral infection^57^. Treatment with an N-BP drug affected the cytokine profile of osteoclasts to a more pro-inflammatory phenotype^56^. The elevation of pro-inflammatory cytokine levels in gingiva following N-BP drug treatment^58^ is thought to be contributed by the immune phenotype-modulated osteoclasts. Therefore, we propose that 800CW-pRIS-derived removal of ZOL in our model attenuated gingival inflammation through the anti-inflammatory function of normalized osteoclasts.

The lack of a specific, efficacious therapy in face of the acute morbidity manifested with BRONJ, notoriously accompanied by severe pain, has generated serious apprehension among patients, leading to decreased compliance of N-BP therapy for bone cancer or osteoporosis^59^. Prevention of BRONJ is of particular concern in conjunction with routine dentoalveolar surgical treatments^60^. Our study indicates that the local intra-oral application of 800CW-pRIS around a tooth to be extracted offers a simple and convenient means to attenuate the effect of N-BP on tooth extraction wound healing. Because intra-oral topical application of 800CW-pRIS-DNV does not distribute to the distant skeletal system (Fig. 9), this approach would also preserve the pharmacological benefit of legacy N-BP targeted to the skeleton, such as appendicular and vertebral bone. This study provides a scientific basis to develop a promising prophylactic approach to solving the scourge of BRONJ without affecting the therapeutic anti-resorptive effect of legacy N-BP.

## Supplementary information

Supplementary Information

Supplementary Data 1

Description of Additional Supplementary Files

Reporting Summary

## Data Availability

The data that support the findings of this study are available from the corresponding author upon reasonable request. Source data for the figures are available as Supplementary Data 1.
