# Supplementary material for: Fluorescent risedronate analogue 800CW-pRIS improves tooth extraction-associated abnormal wound healing in zoledronate-treated mice
Source: Commun Med (Lond). 2022 Sep 5;2:112. doi: 10.1038/s43856-022-00172-x (PMC9445170; doi:10.1038/s43856-022-00172-x)

Supplementary Information

Fluorescent risedronate analogue 800CW-pRIS improves tooth extraction associated abnormal wound healing in zoledronate-treated mice

Hiroko Okawa, Takeru Kondo, Akishige Hokugo, Philip Cherian, Oskar Sundberg, Jesus J. Campagna, Boris A. Kashemirov, Varghese John, Shuting Sun, Frank H. Ebetino, Charles E. McKenna, Ichiro Nishimura

Contact corresponding author:
Ichiro Nishimura
Weintraub Center for Reconstructive Biotechnology
UCLA School of Dentistry
10833 Le Conte Avenue
Los Angeles, CA, USA 90095
Email: inishimura@dentistry.ucla.edu

Fig. S1: MS spectra of intermediate compounds 4 and 5 from the synthesis of 800CW-pRIS

Compound 4

LRMS (+ ve mode): In the positive mode, the parent molecule was not seen but monosodium (479.1 m/z), disodium (501.1 m/z), and trisodium (523.2 m/z) adducts of **4** were observed.

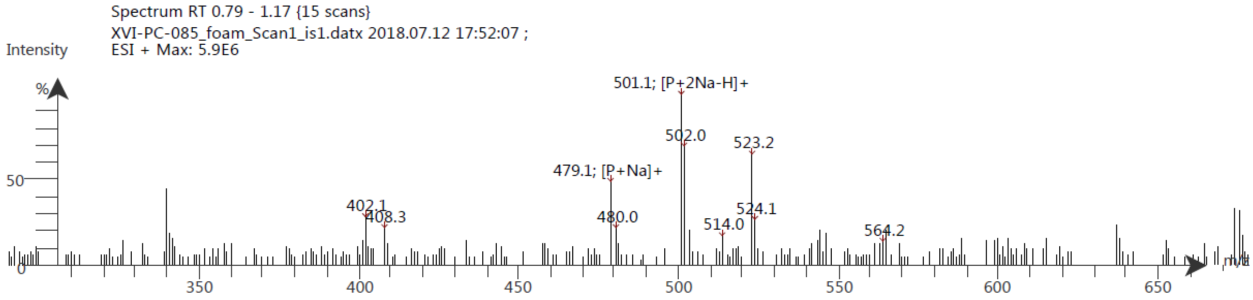

Compound 5

LRMS (+ ve mode): The peak for intermediate **5** was seen at 357.0 m/z in positive mode. Mono and disodium adducts were observed at 379.0 m/z and 400.9 m/z respectively.

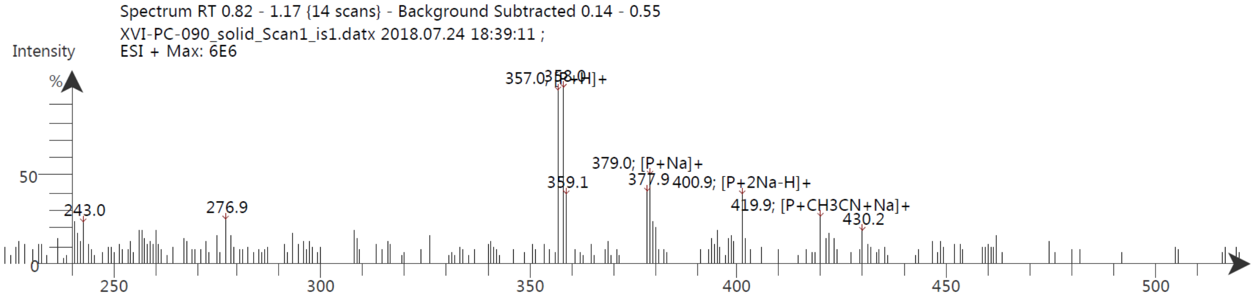

Fig. S2: HPLC purity of 800CW-pRIS

HPLC chromatograph of 800CW-pRIS (Shimadzu HPLC equipped with diode array detector, column: Phenomenex Luna 5 µ C18(2) 100 Å analytical column (250 × 4.6 mm) Mobile phases: A = 0.1M TEAAc/20%MeOH and B = 0.1M TEAAc/70% MeOH Gradient: 0% B (0-7mins), 0-100%B (7 – 25mins), 100%B (25-30 min) Flow rate: 1mL/min).

<Chromatogram>

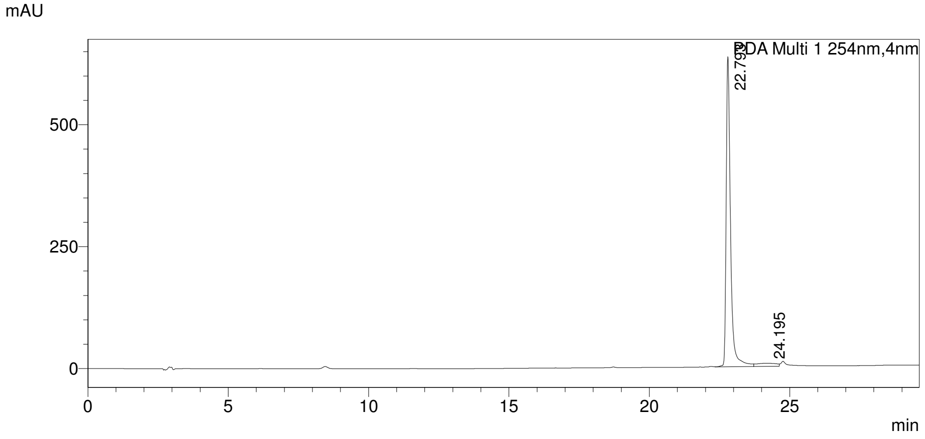

<Peak Table>

PDA Ch1 254nm

Peak#	Ret. Time	Area	Area%
1	22.793	6845801	95.669
2	24.195	309878	4.331
Total		7155679	100.000

Fig. S3: HRMS analysis of 800CW-pRIS (Waters Acquity UPLC H-class Plus combined with XEVO-G2-XS QTOF).

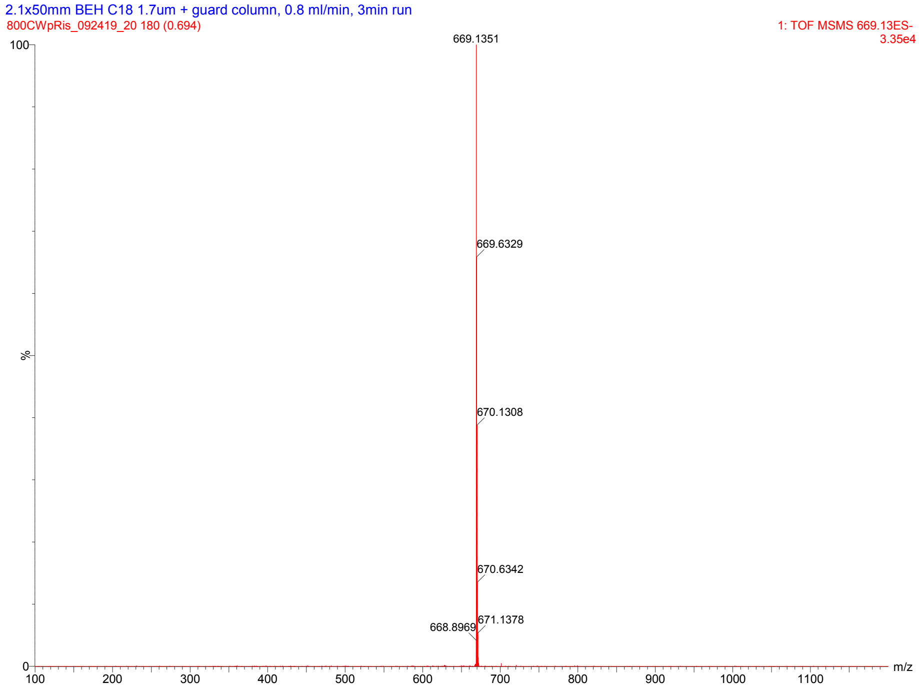

Fig. S4: ^{1}H NMR spectrum of 800CW-pRIS (as triethyl ammonium salt form, spectrum taken in D_2O).

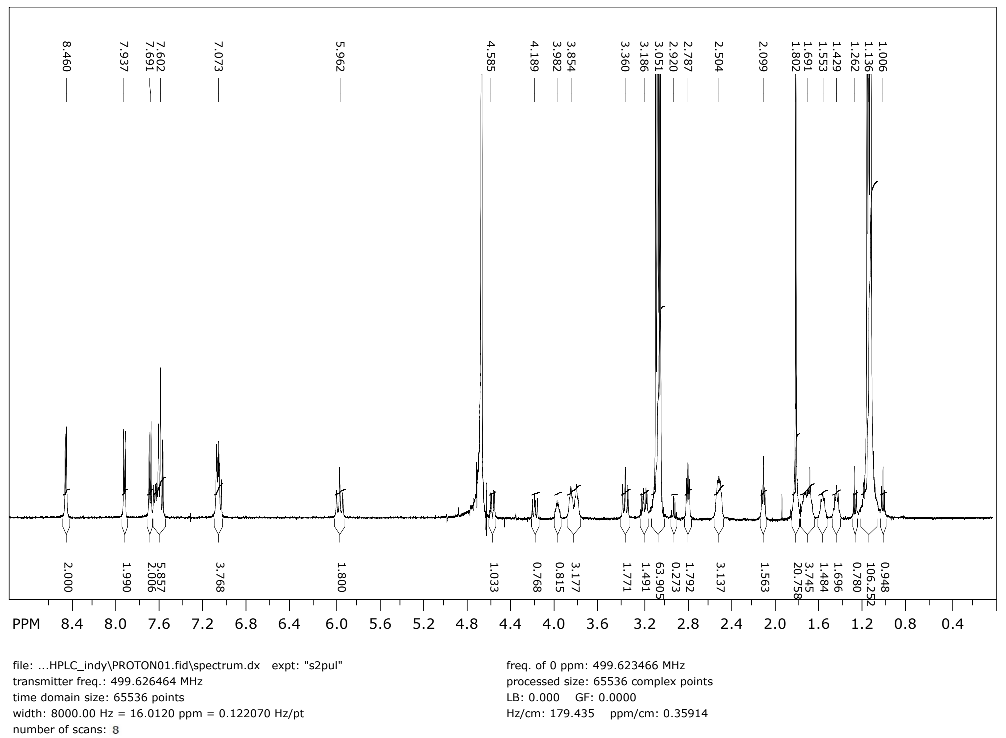

Fig. S5: ^{31}P NMR spectrum of 800CW-pRIS (as triethyl ammonium salt form, spectrum taken in D_2O).

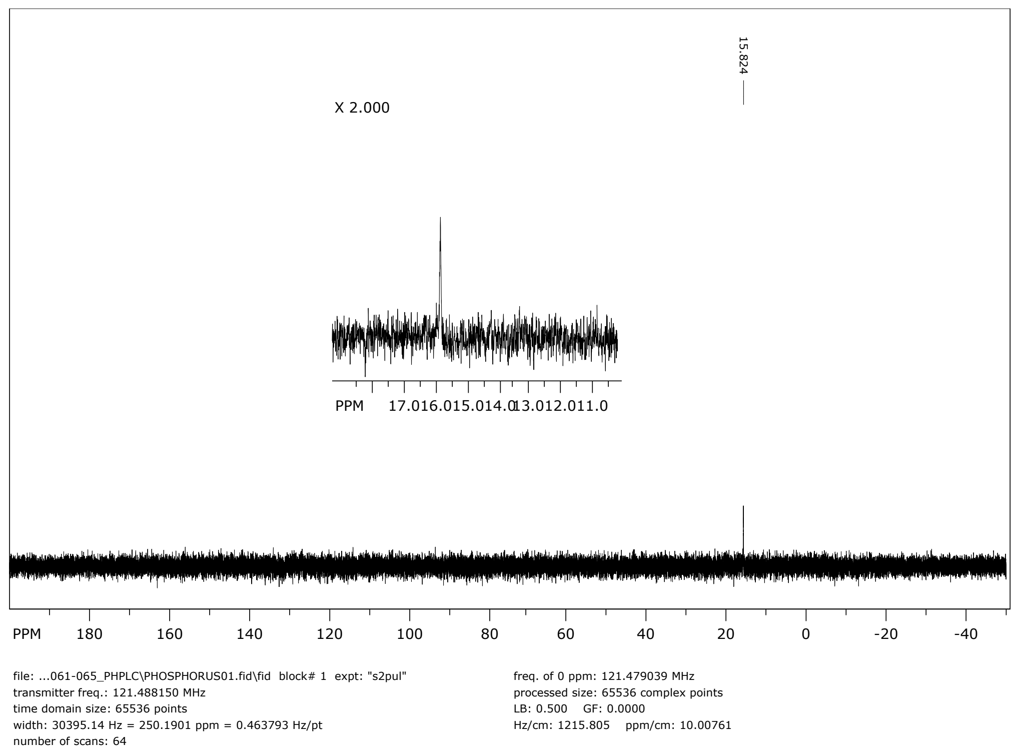

Fig. S6: UV-VIS absorption and fluorescent emission spectra of 800CW-pRIS (spectra were taken in 1X PBS buffer (pH 7.4) and are normalized). The maximum absorption wavelength is 775 nm, and the maximum emission wavelength is 794 nm (slit width for both excitation and emission: 5 nm). The maximum emission wavelength of IRDye 800CW NHS ester in 1X PBS is reported as 792 nm, which is within the possible slit width error (https://www.licor.com/documents/yqbn8ke4zutm01vsnribtdo8e6olx79e)).

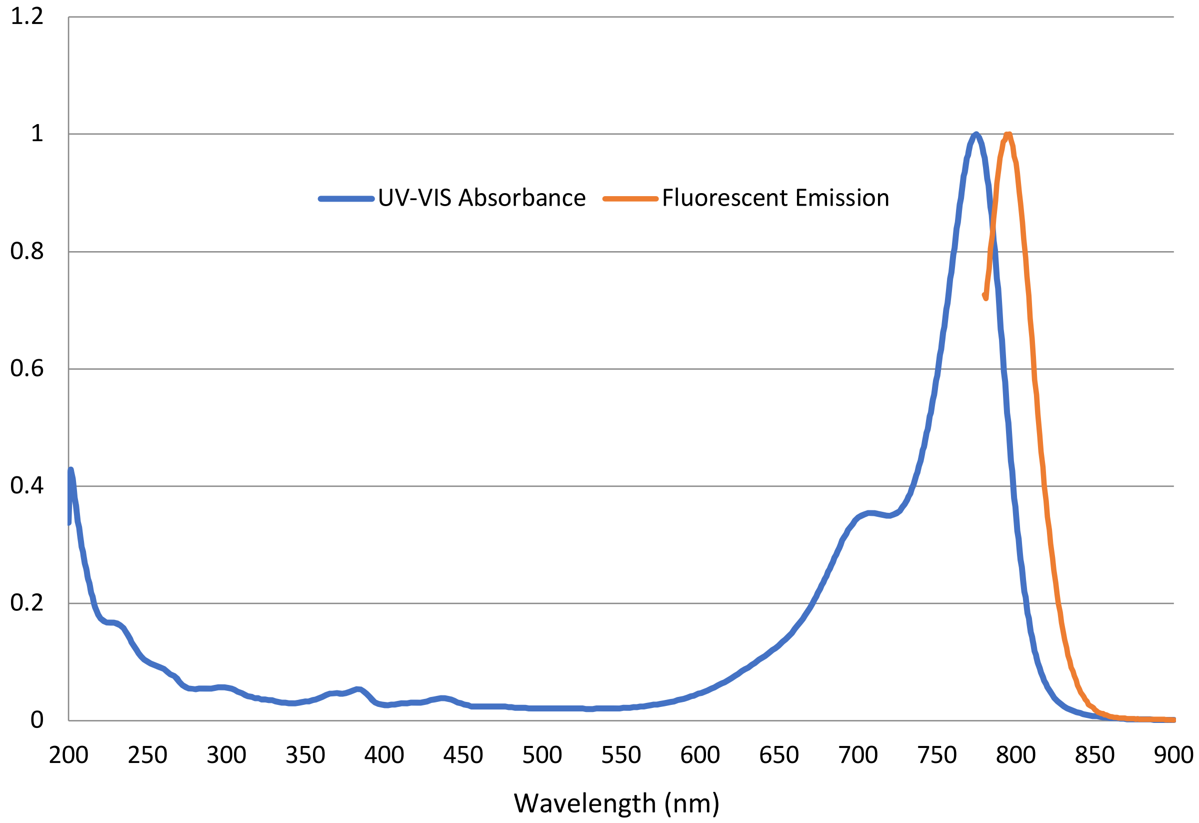

Fig. S7: Gingival swelling associated with tooth extraction of ZOL-pretreated mice

a. Standardized intra-oral photographs demonstrated an abnormal area of palatal gingiva swelling (white arrows) associated with the tooth extraction wound in ZOL-pretreated mice. **b**. The gingival swelling resembled BRONJ lesion. A patient was initially diagnosed as Stage 0 BRONJ after tooth extraction with delayed bone wound healing (white arrow in the dental radiograph) with gingival swelling (white arrows), which later advanced to Stage 1 with fistulation (red arrow). **c**. Mouse palatal histological cross-sections corresponding to panel **a**. The vasculatures (white bracket) in the tooth-extraction side (right side) were less prominent than those of the non-extraction side (left side). In addition to a large area of palatal bone necrosis (Nec), the gingival tissue of the tooth extraction side demonstrated epithelial hyperplasia (Epi), inflammatory cell infiltration (Inf) and gingival connective tissue swelling (Conn). These abnormal gingival responses to tooth extraction in ZOL-pretreated mice compounded to develop an easily identified "gingival swelling" in the standardized intra-oral photographs. As a clinical observation, we measured the "gingival swelling" area, normalized by the circumferential area of the remaining first molar.

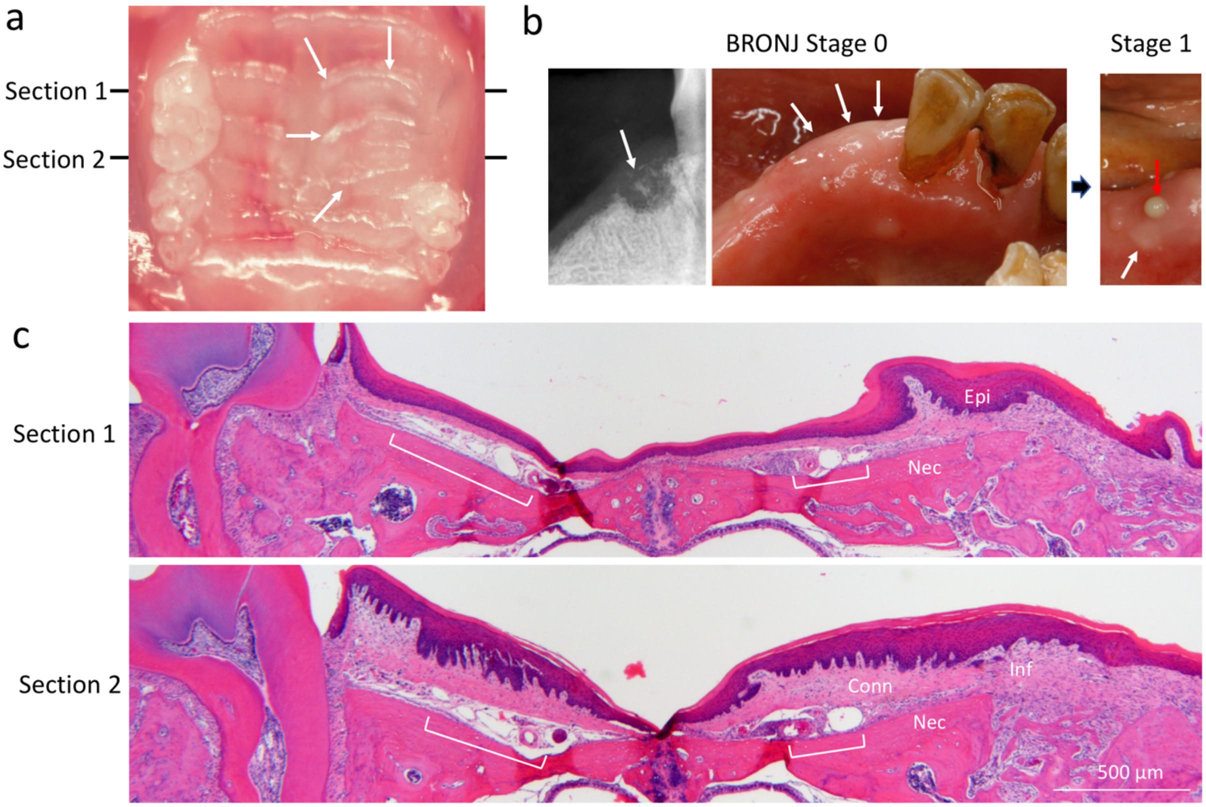

Fig. S8: Computational method of tooth extraction socket bone regeneration using micro-CT.

Step 1: A horizontal image of micro-CT containing the extraction sockets and the corresponding contralateral remaining mesial root, palatal root and distal root were identified.
Step 2: The cross-sectional area of the remaining mesial root, palatal root and distal root was determined.
Step 3: The mirror image of the cross-sectional area of remaining roots was transferred to the tooth extraction side, identifying the “original” tooth extraction socket area.
Step 4: A total of 35 layers covering from the apex of the identified the tooth extraction cross-sectional area were stacked up. The bone volume over tissue volume (BV/TV) in each extraction socket was determined and combined.

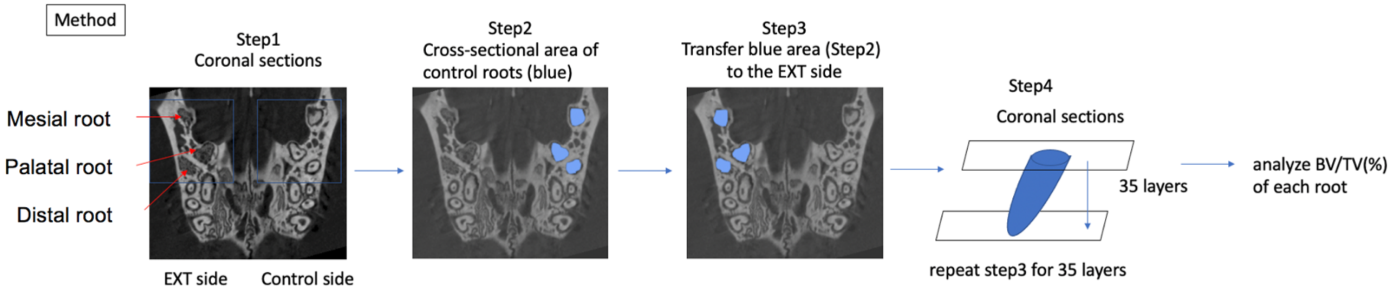

Fig. S9: Inflammation Index.

To determine inflammation index, the extent of inflammatory cell infiltrations within the maxillary gingival tissue was histologically assessed from 0 to 3 by blinded examiners.
Inflammation Index 0: No inflammatory cell infiltration in the palatal gingiva.
Inflammation Index 1: Mild inflammatory cell infiltration, localized on the maxillary bone surface.
Inflammation Index 2: Moderate inflammatory cell infiltration occupying less than 50% area of the palatal gingiva.
Inflammation Index 3: Severe inflammatory cell infiltration occupying more than 50% area of the palatal gingiva.

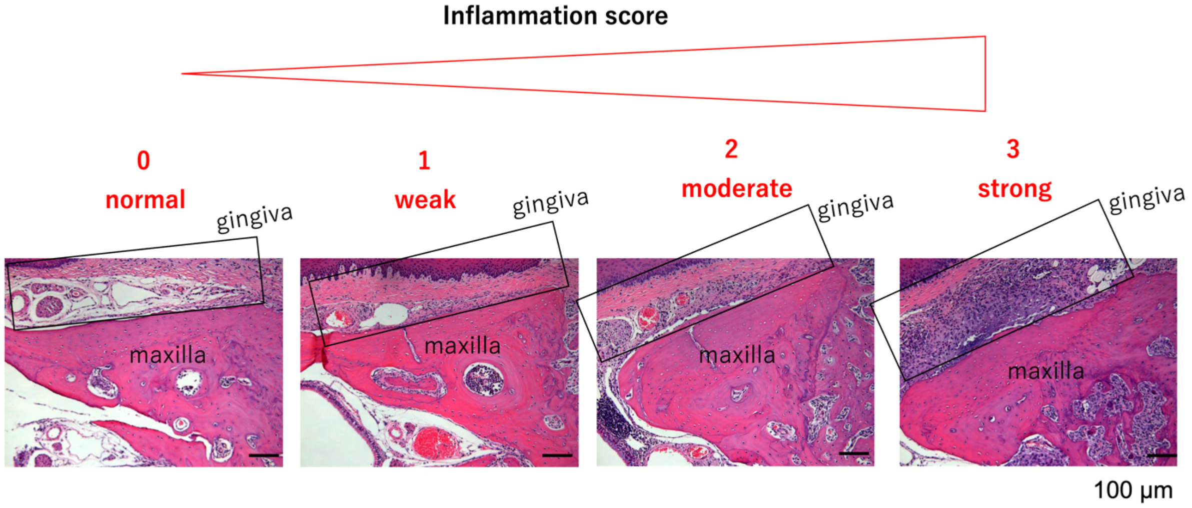

Fig. S10: Docking attempt of 800CW-pRIS to human FPPS (PDB 1YV5).

Structure unable to fit within active site, instead forms interactions at the protein surface. Code; pink structure - 800CW-pRIS carbon backbone; green spheres - Mg^{2+}.

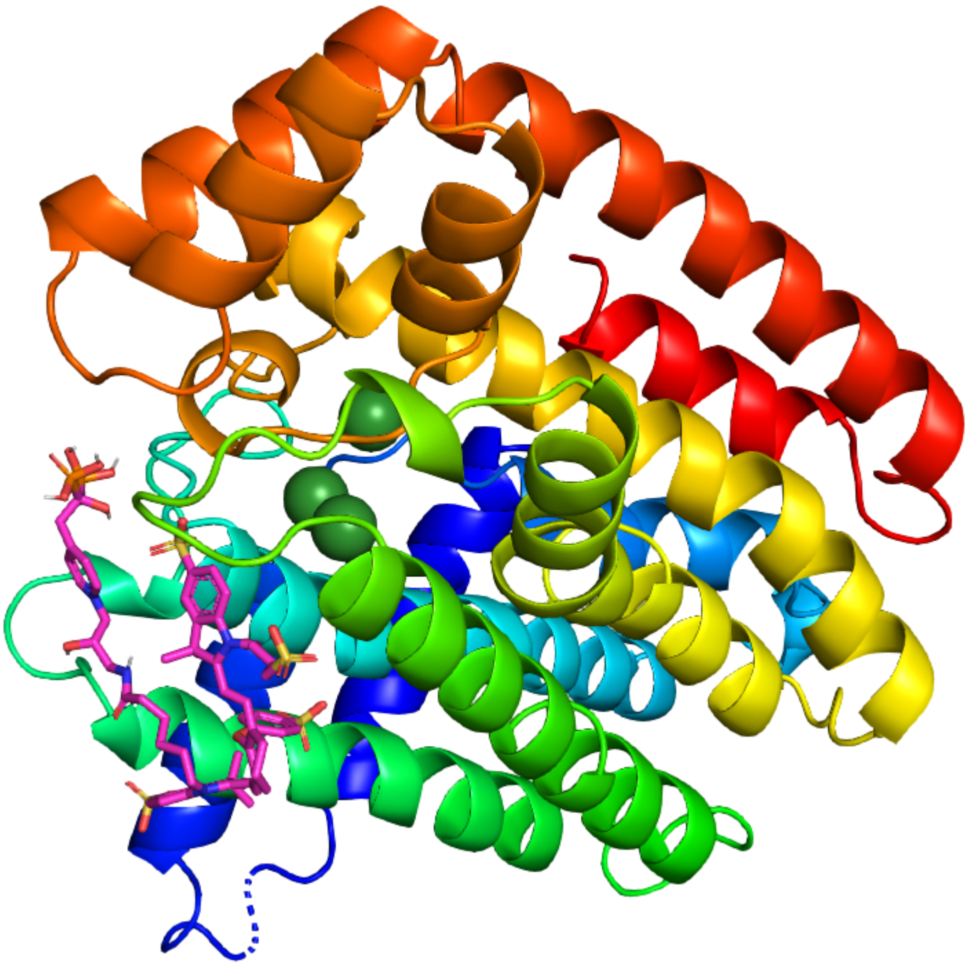

Supplement: Supplementary file 1 — Supplementary Information [file 43856_2022_172_MOESM1_ESM.pdf]
