# Supplementary material for: Fluorescent risedronate analogue 800CW-pRIS improves tooth extraction-associated abnormal wound healing in zoledronate-treated mice
Source: Commun Med (Lond). 2022 Sep 5;2:112. doi: 10.1038/s43856-022-00172-x (PMC9445170; doi:10.1038/s43856-022-00172-x)
Supplement: Supplementary file 3 — Description of Additional Supplementary Files [file 43856_2022_172_MOESM3_ESM.pdf]

Description of Additional Supplementary Files

File Name: Supplementary Data 1

Description: Source data for the figures
